# Synthesis of Metalorganic Copolymers Containing Various Contorted Units and Iron(II) Clathrochelates with Lateral Butyl Chains: Conspicuous Adsorbents of Lithium Ions and Methylene Blue

**DOI:** 10.3390/polym14163394

**Published:** 2022-08-19

**Authors:** Suchetha Shetty, Noorullah Baig, Moustafa Sherief Moustafa, Saleh Al-Mousawi, Bassam Alameddine

**Affiliations:** 1 Department of Mathematics and Natural Sciences, Gulf University for Science and Technology, West Mishref, Kuwait City 32093, Kuwait; 2 Functional Materials Group, Gulf University for Science and Technology, West Mishref, Kuwait City 32093, Kuwait; 3 Department of Chemistry, Kuwait University, Kuwait City 32093, Kuwait

**Keywords:** iron(II) clathrochelate copolymers, poly(vinylene sulfide), poly(vinylene sulfone), one-pot synthesis, click-reaction, methylene blue uptake, lithium ion adsorption

## Abstract

We report the synthesis of three highly soluble metalorganic copolymers, TCP1–3, that were made from a one-pot complexation of iron(II) clathrochelate units that are interconnected by various thioether-containing contorted groups. TCP1–3 were converted into their poly(vinyl sulfone) derivatives OTCP1–3 quantitatively via the selective oxidation of the thioether moieties into their respective sulfones. All of the copolymers, TCP1–3 and OTCP1–3, underwent structural analysis by various techniques; namely, ^1^H- and ^13^C-nuclear magnetic resonance (NMR), Fourier transform infrared (FTIR), X-ray photoelectron spectroscopy (XPS), and gel permeation chromatography (GPC). The copolymers were tested as potent lithium ions adsorbents revealing a maximum adsorption (q_m_) value of 2.31 mg g^−1^ for OTCP2. Furthermore, this same copolymer was found to be a promising adsorbent of methylene blue (MEB); an isothermal adsorption study divulged that OTCP2’s uptake of MEB from an aqueous solution (following the Langmuir model) was, at maximum adsorption capacity, (q_m_) of 480.77 mg g^−1^; whereas the kinetic study divulged that the adsorption follows pseudo second-order kinetics with an equilibrium adsorption capacity (q_e,cal_) of 45.40 mg g^−1^.

## 1. Introduction

Click reactions have proven to be very useful in making a wide variety of functional materials as they afford the desired compounds in high yields from readily accessible reagents under mild reaction conditions and without requiring complex purification techniques since they do not generate many by-products [1,2]. Therefore, click chemistry has been increasingly employed for various applications, such as pharmaceuticals [3], cancer theranostics [4], nanomaterials [5], macrocyles [6], and polymers [7]. Thiol-based click reactions have gained growing interest and myriad chemistries have been developed; namely, thiol-ene and -yne [7,8], thiol-epoxy [9], thiol-isocyanate [10], and thiol-Michael addition reactions [11]. Among others, the thiol-yne click reactions are widely used in the synthesis of macromolecules for various applications [8,12,13]; for example, the synthesis of conjugated polymers [14], wherein electron-rich sulfur atoms tether the aromatic comonomers, has led to an improved level of electron mobility [15,16]. The sulfide units offer another advantage by allowing for their versatile oxidation into sulfones [17,18], thus affording highly polar polymers [19,20] which find applications in fuel cells [21,22,23,24], organic light emitting diodes (OLEDs) [25,26], chemosensors [27], proton exchange membranes [28], and gas separation membranes [29].

The prevailing interest in lithium purification for the past two decades is mainly due to its extensive applications in cutting edge technologies, including batteries, catalysis, and nuclear energy [30,31]. Furthermore, lithium, the natural sources of which are scarce, is considered an essential component of future energy sources due to its superior electrochemical and physical properties when compared to other metals [32,33]. Hence, there have been growing numbers of attempts to extract lithium from brine and recycle it from used devices using myriad separation processes, such as solvent extraction, precipitation, electrochemistry, and adsorption. The latter of these methods is considered to be very promising, especially for the large-scale separation of lithium, because of the several advantages it represents, namely its versatility, eco-friendliness, and cost-effectiveness [34,35,36].

The pollution of fresh water resources has become a major environmental challenge due to the rapid rate of industrialization and the world’s growing population as the major contaminants which constitute a potential health threat to living organisms encompass dyes and pigments, heavy metal ions, organic solvents, antibiotics, and microbial species [37,38,39,40]. Organic dyes are the chief contributors to water pollution, this is caused by their discharge from various industrial sectors, such as those which handle fabric, plastic, printing, photography, paper-pulp, paint, and leather [41,42,43,44,45,46]. Methylene blue (MEB), a thiazine dye, is a common aromatic cationic dye which is carcinogenic [47,48] besides also causing various other health complications [49,50,51,52]. The removal of MEB from wastewater is thus critical and various techniques have been developed for this purpose, namely coagulation, sedimentation, chemical treatment, membrane filtration, and adsorption [53,54,55,56,57]. The latter of these is considered to be the most promising because it offers many upsides, mainly the versatile synthesis of various adsorbent materials and their efficacy [58,59,60,61].

Numerous clathrochelate complexes which bear a central metal ion that is encapsulated in three-dimensional organic bidentate ligands have been synthesized and tested as biosensors [62,63], catalysts for hydrogen generation [64,65], porous polymer adsorbents [66], semiconducting materials [67], organogels [68], and supramolecules [69,70,71,72] as well as metalorganic frameworks for gas separation and purification [73,74]. Recently, we have reported the synthesis of porous copolymers bearing iron(II) clathrochelate units that disclose prominent dye adsorption properties [75,76]. In this work, we explore the synthesis of three new thioether-containing iron(II) clathrochelate copolymers, **TCP1–3**, that were made from a one-pot synthesis process and which underwent subsequent selective oxidation into their sulfone copolymer derivatives **OTCP1–3** before testing all of the copolymers as potent adsorbents. This testing revealed a maximum adsorption value of 2.31 mg g^−1^ and MEB removal properties reaching ~481 mg g^−1^ from an aqueous solution.

## 2. Materials and Methods

The chemical reactions reported herein were carried out under a positive flow of dry argon. The required 4-Mercaptophenylboronic acid and FeCl_2_ were purchased from Merck (Darmstadt, Germany) while the butyl dioxime, 1,4-diethynyl-9,10-dihydro-9,10-[1,2] benzenoanthracene **1**, 2,8-diethynyl-4,10-dimethyl-6,12-dihydro-5,11-methanodibenzo[b,f][1,5]diazocine **2,** and 2,7-diethynyl-9,9-dimethyl-9H-fluorene **3** were synthesized according to the procedures that have been reported in the literature [76,77,78,79]. The chemical reagents that were purchased from Merck (Darmstadt, Germany) and HiMedia (Maharashtra, India) were used without further purification unless otherwise specified. The tetrahydrofuran (THF), dichloromethane (DCM), chloroform, acetone, diethyl ether, methanol, and hexane were dried over molecular sieves and deoxygenated by bubbling with argon for 30 min. The thin-layer chromatography (TLC) was done using silica gel 60 F254 deposited on aluminum sheets and revealed using a UV lamp. The NMR (^1^H: 600 MHz, ^13^C:150 MHz) spectra were recorded on a Bruker BioSpin GmbH 600 MHz spectrometer using CD_2_Cl_2_ and DMSO-d_6_ as solvents and tetramethylsilane (TMS) as the internal reference with the chemical shifts (δ) given in ppm. The FT-IR spectra were recorded on an Agilent Cary 630 FTIR instrument. The thermogravimetric analysis (TGA) was recorded on a Shimadzu TGA-60H (Kyoto, Japan) analyzer in order to measure the thermal stability of the analytes up to 800 °C using a heating rate of 10 °C/min under an inert atmosphere of pure nitrogen. The X-ray photoelectron spectroscopy (XPS) data were recorded with a Thermo ESCALAB 250 Xi using a monochromatic Al Kα-radiation source (1486.6 eV) with a spot size of 850 μm. The spectral acquisition and processing were performed by employing Thermo Advantage software (Version 4.87). The base pressure in the XPS analysis chamber ranged between 10^−10^ and 10^−9^ torr. The analyzer was operated with a pass energy of 20 eV, dwell time of 50 min, and with a step size of 0.1 eV. Thermo (DFS) with a standard PFK (per-fluorokerosene) as its lock mass was used to obtain the electron impact high-resolution mass spectra (EI-HRMS). X-Calibur accurate mass calculation software was employed in order to convert the analyzed data to an accurate mass. The concentrations of Li^+^ before and after the adsorption by the polymers were recorded using an inductively coupled plasma mass spectrometry (ICP-MS) instrument (Perkin-Elmer, Nexion 2000P). The molecular weights of the copolymers were determined using an Agilent 1260 infinity II gel permeation chromatograph (GPC) that was equipped with a refractive index (RI) detector using two columns (PL mixed-C), which were calibrated with twelve monodisperse polystyrene (PS) standards, and employing THF as an eluent at a flow rate of 1.0 mL min^−1^ at room temperature.

### 2.1. Synthesis

#### 2.1.1. Synthesis of TC1 (Procedure A)

A Schlenk tube was charged with 1,4-diethynyl-9,10-dihydro-9,10-[1,2] benzenoanthracene **1** (0.2 g, 0.67 mmol, 1 eq.) and 4-mercaptophenylboronic acid (0.204 g, 1.32 mmol, 2 eq.) in a THF (10 mL). The reaction mixture was purged with argon and stirred overnight at 50 °C. The resulting solution was precipitated using hexane, affording a white solid (0.39 g, 96%) after filtration. ^1^H-NMR (600 MHz, DMSO-d_6_, ppm): δ 8.13 (*brs*, 4H, -OH), 7.84 (*brm*, 4H, ArH), 7.53–7.48 (*m*, 8H, ArH), 7.46–7.42 (*m*, 6H, ArH), 7.02–7.01 (*brs*, 2H, Vinylic-CH), 6.94–6.93 (*brm*,2H, vinylic-CH), 5.98 (*s*, 2H, triptycene-CH); ^13^C-NMR (150 MHz, DMSO-d_6_, ppm): δ 144.32, 145.44, 137.63, 135.60, 130.80, 128.42, 127.90, 125.58, 124.36, 117.06, 49.58; EI-HRMS: m/z calculated for (M^•+^) C_30_H_28_B_2_O_4_S_2_ 610.1615 found 610.1609.

#### 2.1.2. Synthesis of TC2

TC2 was prepared according to the method of Procedure A with 2,8-diethynyl-4,10-dimethyl-6,12-dihydro-5,11-methanodibenzo[b,f][1,5]diazocine **2** (0.2 g, 0.67 mmol, 1 eq.) and 4-mercaptophenylboronic acid (0.206 g, 1.34 mmol, 2 eq.) in THF (10 mL). White solid (0.39 g, 95%); ^1^H-NMR (600 MHz, DMSO-d_6_, ppm): δ 8.06 (*brs*, 4H, -OH), 7.80–7.78 (*m*, 4H, ArH), 7.77–7.76 (*m*, 4H, ArH), 6.99 (*s*, 2H, ArH), 6.87 (*s*, 2H, ArH), 6.64 (*br*,2H, vinylic-CH), 6.55 (*br*, 2H, vinylic-CH), 4.53 (*d*, 2H, *J* = 12 Hz, methylene-CH), 4.26 (*s*, 2H, methylene-CH), 3.97 (*d*, 2H, *J* = 12 Hz, methylene-CH), 2.18 (s, 6H, methyl-CH); ^13^C-NMR (150 MHz, DMSO-d_6_, ppm): δ 145.63, 139.15, 135.03, 129.06, 128.01, 127.34, 126.63, 124.89, 123.64, 122.50, 119.62, 66.99, 54.37, 21.01; EI-HRMS: m/z calculated for (M^•+^) C_33_H_32_B_2_N_2_O_4_S_2_ 606.1990 found 606.1984.

#### 2.1.3. Synthesis of TC3

TC3 was prepared according to the method of Procedure A with 2,7-diethynyl-9,9-dimethyl-9H-fluorene **3** (0.2 g, 0.83 mmol, 1 eq.) and 4-mercaptophenylboronic acid (0.254 g, 1.65 mmol, 2 eq.) in THF (12 mL). White solid (0.42 g, 91%); ^1^H-NMR (600 MHz, DMSO-d_6_, ppm): δ 8.47 (*brs*, 4H, -OH), 7.82–7.68 (*m*, 8H, ArH), 7.49–7.42 (*m*, 6H, ArH), 7.34 (*brm*,2H, vinylic-CH), 6.81 (*brm*, 2H, Vinylic-CH), 1.48 (*s*, 6H, methyl-CH_3_); ^13^C-NMR (150 MHz, DMSO-d_6_, ppm): δ 148.82, 137.34, 135.11, 133.58, 132.72, 127.59, 125.96, 123.19, 120.24, 116.65, 46.35, 26.88; EI-HRMS: m/z calculated for (M^•+^) C_31_H_28_B_2_O_4_S_2_ 550.1615 found 550.1610.

#### 2.1.4. Synthesis of TCP1 (Procedure B)

**TC1** (0.3 g, 0.49 mmol, 1 eq.), butyl dioxime (0.295 g, 1.47 mmol, 3 eq.), and iron(II) chloride (0.06 g, 0.49 mmol, 1 eq.) in chloroform (10 mL) were charged in a Schlenk tube under argon and refluxed for 48 h. The reaction mixture was dried by evaporating the solvent under reduced pressure and the resulting residue was extracted with DCM from the aqueous solution. The combined organic layer was washed with deionized H_2_O (100 mL X 3), concentrated, and hexane was added to precipitate the product which was isolated by filtration under vacuum and washed successively with hexane (20 mL), acetone (20 mL), and methanol (20 mL). Red solid (0.50 g, 86%); ^1^H-NMR (600 MHz, DMSO-d_6_, ppm): *δ* 7.66 (*brs*, 4H, ArH), 7.47 (*brs*, 10H, ArH), 7.00 (*brs*, 4H, ArH), 6.88–6.87 (*br*, 4H, vinylic-CH), 6.02 (*s*, 2H, triptycene-CH), 2.79 (*brs*, 12H, butyl-CH_2_), 1.49 (*brs*, 12H, butyl-CH_2_), 1.39 (*brs*, 12H, butyl-CH_2_), 0.88–0.86 (*t*, 18H, *J* = 12 Hz, butyl-CH_3_); ^13^C-NMR (150 MHz, DMSO-d_6_, ppm): δ 155.90, 144.93, 142.67, 132.31, 130.50, 129.00, 128.73, 128.32, 126.08, 125.07, 123.85, 116.34, 49.83, 28.24, 22.85, 22.47, 13.79; FTIR (KBr, cm^−1^): 3055 (aromatic =CH stretch.), 2958 (aliphatic -C-H stretch.), 1670 (C=N stretch.), 1592 (C=C stretch.), 1464 (aliphatic -C-H bend.), 818 (aliphatic -C-H bend.), and 701 (aliphatic C=C bend.); GPC (THF): M_w_ (g mol^−1^): 30198, M_n_ (g mol^−1^): 10066, Đ: 3.0.

#### 2.1.5. Synthesis of TCP2

TCP2 was prepared according to the method of Procedure B with **TC2** (0.2 g, 0.33 mmol, 1 eq.), butyl dioxime (0.198 g, 0.99 mmol, 3 eq.), and iron(II) chloride (0.04 g, 0.33 mmol, 1 eq.) in chloroform (6.5 mL). Red solid (0.33 g, 83%); ^1^H-NMR (600 MHz, DMSO-d_6_, ppm): *δ* 7.58 (*brm*, 4H, ArH), 7.33 (*brm*, 4H, ArH), 7.24 (*brs*, 2H, ArH), 7.01 (*brm*, 4H, vinylic-CH), 6.66 (*brm*, 2H, ArH), 4.56 (*d*, 2H, *J* = 12 Hz, methylene-CH), 4.34 (*s*, 2H, methylene-CH), 4.01 (*d*, 2H, *J* = 12 Hz, methylene-CH), 2.79 (*brs*, 12H, butyl-CH_2_), 2.36 (*brs*, 6H, methyl-CH) 1.49 (*brs*, 12H, butyl-CH_2_), 1.25 (*brs*, 12H, butyl-CH_2_), 0.81 (*brs*, 18H, butyl-CH_3_); ^13^C-NMR (150 MHz, DMSO-d_6_, ppm): δ 153.49, 142.95, 135.22, 132.89, 131.01, 128.55, 128.34, 112.38, 70.72, 59.70, 29.03, 26.85, 22.13, 17.27, 14.18; FTIR (KBr, cm^−1^): 3050 (aromatic =CH stretch.), 2958 (aliphatic -C-H stretch.), 1684 (C=N stretch.), 1592 (C=C stretch.), 1464 (aliphatic -C-H bend.), 805 (aliphatic -C-H bend.), and 739 (aliphatic C=C bend.); GPC (THF): M_w_ (g mol^−1^): 21404, M_n_ (g mol^−1^): 7493, Đ: 2.8.

#### 2.1.6. Synthesis of TCP3

TCP3 was prepared according to the method of Procedure B with TC3 (0.2 g, 0.36 mmol, 1 eq.), butyl dioxime (0.155 g, 1.09 mmol, 3 eq.), and iron(II) chloride (0.046 g, 0.36 mmol, 1 eq.) in chloroform (7.3 mL). Red solid (0.36 g, 88%); ^1^H-NMR (600 MHz, DMSO-d_6_, ppm): *δ* 7.82–7.79 (*brm*, 4H, ArH), 7.64 (*brm*, 4H, ArH), 7.49 (*brm*, 4H, ArH), 7.42 (*brm*, 2H, ArH) 7.35 (*brm*, 2H, vinylic-CH), 6.85 (*brm*, 2H, vinylic-CH), 2.80 (*brs*, 12H, butyl-CH_2_), 1.49 (*brs*, 18H, -CH_2_), 1.26 (*brs*, 12H, -CH_2_) 0.83 (*brs*, 18H, butyl-CH_3_); ^13^C-NMR (150 MHz, DMSO-d_6_, ppm): δ 157.83, 145.66, 137.95, 135.50, 133.01, 132.76, 127.61, 126.32, 125.14, 121.22, 118.63, 46.18, 29.03, 27.29, 26.89, 22.15, 14.20; FTIR (KBr, cm^−1^): 3055 (aromatic =CH stretch.), 2967 (aliphatic -C-H stretch.), 1688 (C=N stretch.), 1595 (C=C stretch.), 1468 (aliphatic -C-H bend.), 813 (aliphatic -C-H bend.), and 734 (aliphatic C=C bend.); GPC (THF): M_w_ (g mol^−1^): 21061, M_n_ (g mol^−1^): 6315, Đ: 3.3.

#### 2.1.7. Synthesis of OTCP1 (Procedure C)

A solution of aqueous H_2_O_2_ (8 mL, 30 wt%) was added, dropwise, to a stirring suspension of TCP1 (0.1 g, 0.084 mmol) in acetic acid (6 mL) and the reaction mixture was heated at 50 °C for 1 h. The resulting red precipitate was filtered off and washed with deionized H_2_O (20 mL) and diethyl ether (20 mL) then dried under reduced pressure. Red solid (0.1 g, 96%).^1^H-NMR (600 MHz, DMSO-d_6_, ppm): *δ* 8.01 (*brm*, 8H, ArH), 7.54 (*brm*, 8H, ArH), 7.02 (*brs*, 6H, ArH and vinylic-CH), 5.95 (*s*, 2H, triptycene-CH), 2.79 (*brs*, 12H, butyl-CH_2_), 1.48 (*brs*, 12H, butyl-CH_2_), 1.22 (*brs*, 12H, butyl-CH_2_), 0.79 (*brs*, 18H, butyl-CH_3_); ^13^C-NMR (150 MHz, DMSO-d_6_, ppm): δ 157.49, 146.35, 144.29, 135.10, 132.12, 130.42, 129.97, 126.15, 125.42, 124.11, 123.53, 116.00, 48.20, 28.47, 26.34, 21.56, 13.59; FTIR (KBr, cm^−1^): 3055 (aromatic =CH stretch.), 2958 (aliphatic -C-H stretch.), 1670 (C=N stretch.), 1592 (C=C stretch.), 1464 (aliphatic -C-H bend.), 1314 (S=O stretch.), 1120 (S=O stretch), 818 (aliphatic -C-H bend.), and 701 (aliphatic C=C bend.); GPC (THF): M_w_ (g mol^−1^): 20017, M_n_ (g mol^−1^): 7056, Đ: 2.8.

#### 2.1.8. Synthesis of OTCP2

OTCP2 was prepared following the method of Procedure C with TCP2 (0.1 g, 0.083 mmol), acetic acid (6 mL), and 30 wt% aq. H_2_O_2_ (8 mL). Red solid (0.1 g, 95%).^1^H-NMR (600 MHz, DMSO-d_6_, ppm): *δ* 7.79 (*brs*, 8H, ArH), 7.65–7.54 (*brm*, 4H, ArH), 7.33 (*brs*, 4H, vinylic-CH), 5.03 (*brd*, 2H, methylene-CH), 4.52 (*brm*, 4H, methylene-CH), 2.76 (*brs*, 12H, butyl-CH_2_), 2.27 (brm, 6H, methyl-CH) 1.49 (*brs*, 12H, butyl-CH_2_), 1.23 (*brs*, 12H, butyl-CH_2_), 0.77 (*brs*, 18H, butyl-CH_3_); ^13^C-NMR (150 MHz, DMSO-d_6_, ppm): δ 158.30, 146.82, 135.57, 130.94, 129.60, 127.70, 126.33, 124.75, 117.11, 71.76, 60.05, 29.03, 26.86, 22.11, 17.22, 14.14; FTIR (KBr, cm^−1^): 3050 (aromatic =CH stretch.), 2958 (aliphatic -C-H stretch.), 1684 (C=N stretch.), 1592 (C=C stretch.), 1464 (aliphatic -C-H bend.), 1314 (S=O stretch.), 1120 (S=O stretch.) 805 (aliphatic -C-H bend.), and 739 (aliphatic C=C bend.). 

#### 2.1.9. Synthesis of OTCP3 

OTCP3 was prepared following the method of Procedure C with TCP3 (0.1 g, 0.088 mmol), acetic acid (6 mL), and 30 wt% aq. H_2_O_2_ (8.5 mL). Red solid (0.104 g, 98%).^1^H-NMR (600 MHz, DMSO-d_6_, ppm): *δ* 7.90 (*brm*, 16H, ArH and vinylic-CH), 6.97 (*brm*, 2H, vinylic-CH), 2.80 (*brs*, 12H, butyl-CH_2_), 1.48 (*brs*, 18H, -CH_2_), 1.24 (*brs*, 12H, -CH_2_), 0.81 (*brs*, 18H, butyl-CH_3_); ^13^C-NMR (150 MHz, DMSO-d_6_, ppm): δ 158.90, 146.10, 144.50, 139.22, 132.96, 130.45, 128.34, 127.14, 124.81, 122.58, 116.60, 46.49, 29.03, 26.90, 22.11, 14.17, 14.20; FTIR (KBr, cm^−1^): 3055 (aromatic =CH stretch.), 2967 (aliphatic -C-H stretch.), 1688 (C=N stretch.), 1595 (C=C stretch.), 1468 (aliphatic -C-H bend.),1311(S=O stretch.), 1121(S=O stretch.) 813 (aliphatic -C-H bend.), and 734 (aliphatic C=C bend.).

## 3. Results

### 3.1. Synthesis of Synthons TC1–3

**Scheme 1:**
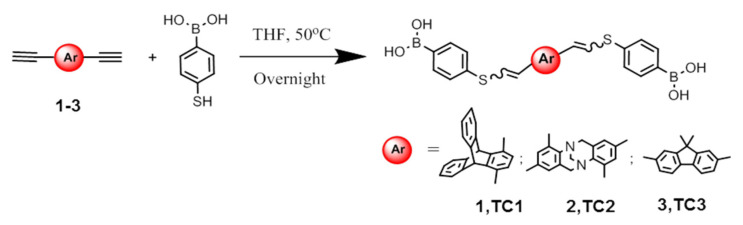
Synthesis of synthons **TC1–3**.

Scheme 1 illustrates the thiol-yne click reaction conditions that were employed in order to synthesize the three diboronic acid synthons **TC1–3** which were made by reacting each of the diethynyl starting materials **1–3** with 4-mercaptophenylboronic acid in THF at 50 °C overnight under argon. It is worth mentioning that nuclear magnetic resonance (^1^H- and ^13^C-NMR) and electron impact high resolution mass spectrometry (EI-HRMS) of the resulting products **TC1–3** confirmed their formation in high purity (Figures S1–S3, S10–S12 and S19–S21 in the Supplementary Materials). 

### 3.2. Synthesis of Copolymers TCP1–3

**Scheme 2:**
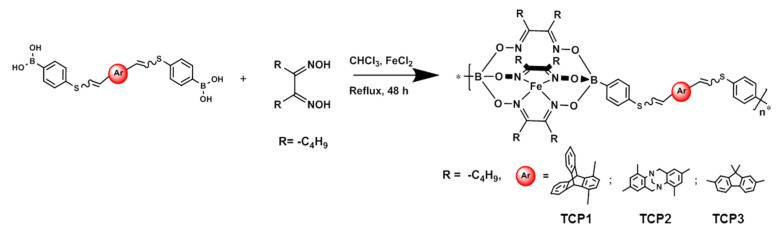
Synthesis of copolymers **TCP1–3**.

**Figure 1:**
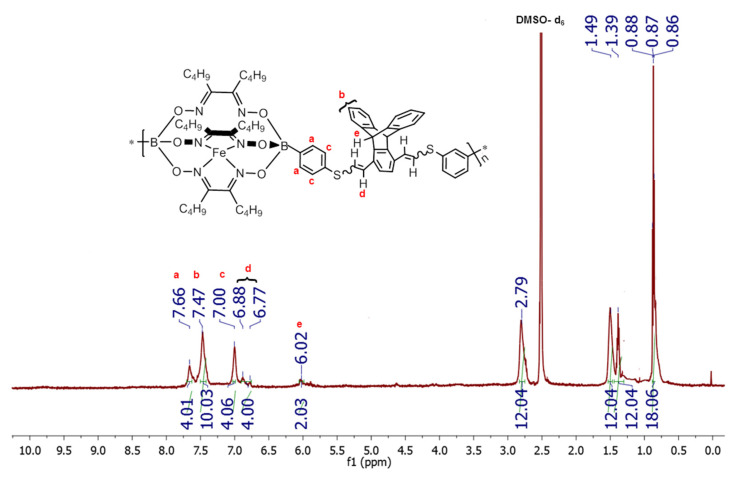
^1^H-NMR spectrum of **TCP1**.

**Figure 2:**
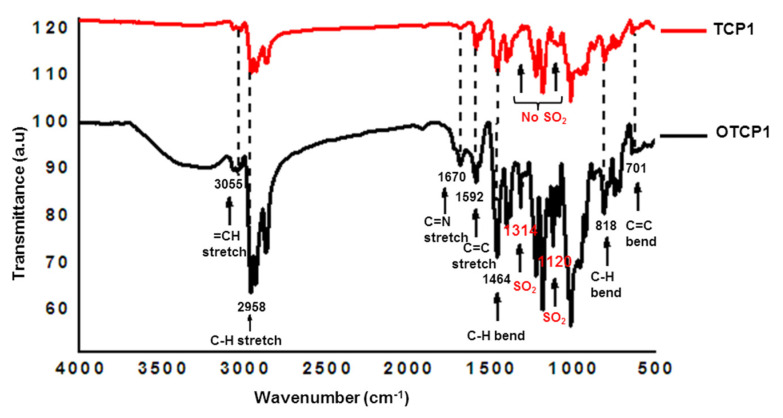
Comparative FTIR spectrum of **TCP1** (**up**) and **OTCP1** (**down**).

**Figure 3:**
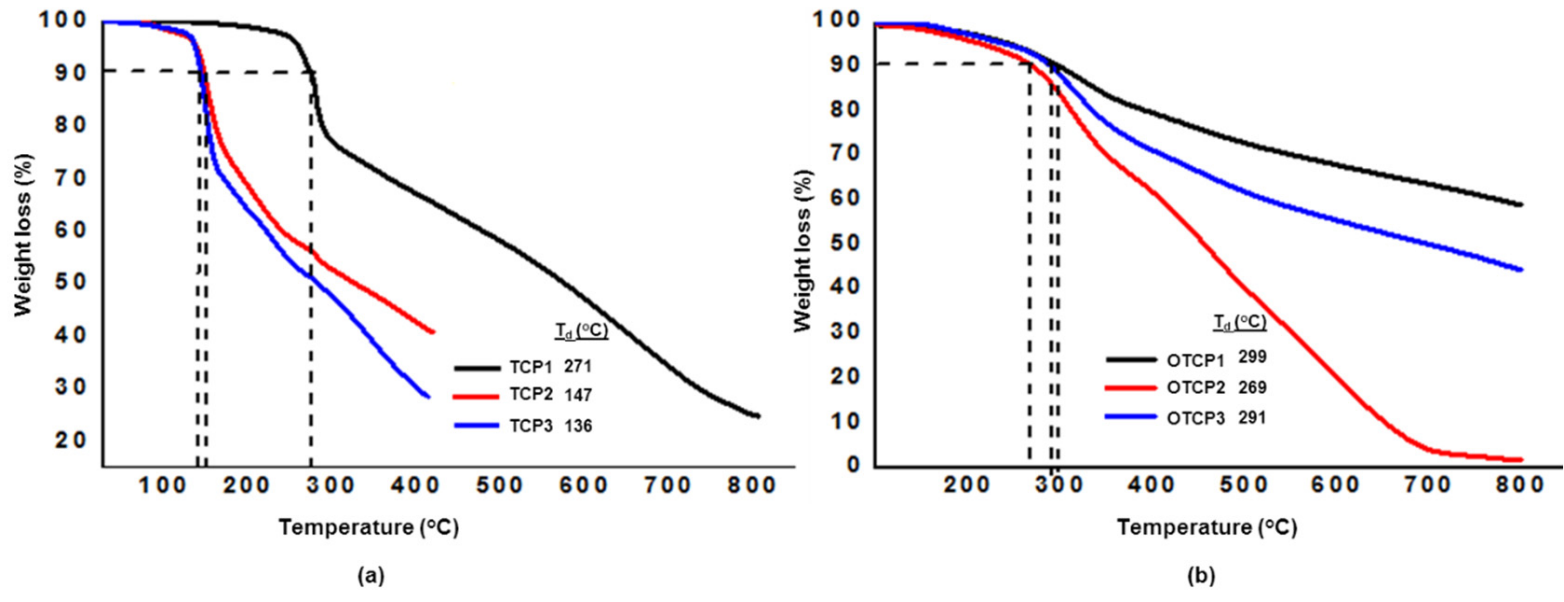
TGA thermograms of copolymers **TCP1–3** (**a**) and **OTCP1–3** (**b**), T_d_ represents the temperature of 10% weight loss.

**Figure 4:**
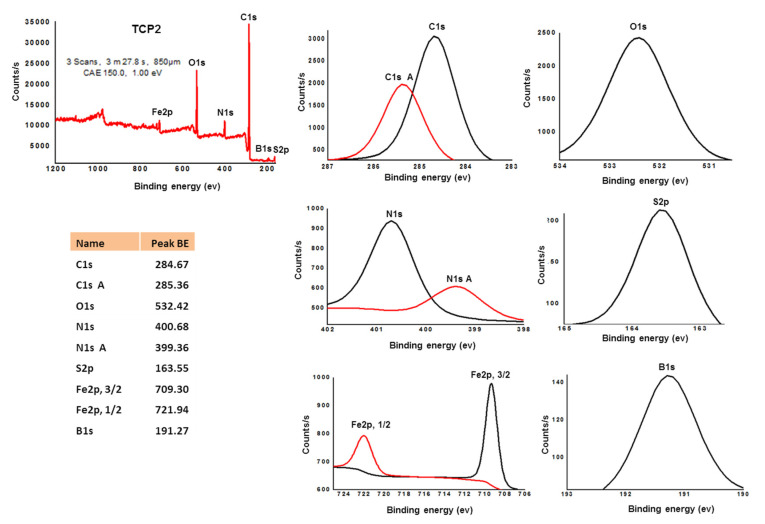
High-resolution survey scan XPS spectra of C1s, O1s, N1s, S2p, Fe2p, and B1s of **TCP2**.

Scheme 2 reveals the reaction of an equimolar amount of FeCl_2_ with each of the synthons **TC1–3** and three equivalents of butyl dioxime in refluxing chloroform under argon for 48 h which afforded the desired iron(II) clathrochelate copolymers **TCP1–3** in high yields (83–88%). The presence of six butyl groups per iron(II) clathrochelate unit bestowed the copolymers with high solubility in various organic solvents such as DCM, chloroform, THF, and DMF, therefore allowing for their thorough structural analysis by ^1^H- and ^13^C- NMR besides the determination of the target copolymers’ molar mass by gel permeation chromatography (GPC). In addition, **TCP1–3** were further analyzed by FTIR, TGA and XPS (Figure 1, Figure 2, Figure 3 and Figure 4, Figures S4–S6, S13–S15, S22–S27 and S31–S33 in the Supplementary Materials).

### 3.3. Synthesis of Copolymers OTCP1–3

**Scheme 3:**
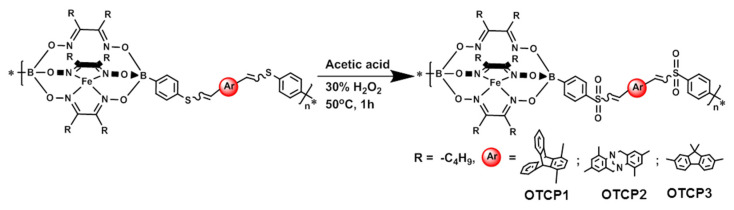
Synthesis of copolymers **OTCP1–3**.

The thioether groups of copolymers **TCP1–3** underwent complete oxidation into their corresponding sulfone moieties. These transformations were achieved by reacting the former compounds with hydrogen peroxide in acetic acid at 50 °C for 1 h [80], which afforded the sulfone-containing copolymers **OTCP1–3** (Scheme 3) in ~96% yield. The target copolymers were found to be sparingly soluble in DCM, chloroform, and THF. Their structures were confirmed by ^1^H- and ^13^C-NMR FTIR and XPS spectroscopy, besides determining the molar mass of **OTCP1** by GPC since it is the only derivative which was soluble enough for this analytical technique (Figure 2, Figures S7–S9, S16–S18, S22–S24, S28–S30 and S33 in the Supplementary Materials). 

Figure 1 illustrates the ^1^H-NMR spectrum of **TCP1**, where the chemical shifts at 7.66 ppm and 7.00 ppm (i.e., peaks a and c) are attributed to the eight aromatic protons of the thiophenyl boronate unit, whereas the ten aromatic protons that were detected at 7.47 ppm (c.f., Figure 1, peak b) are assigned to the triptycene unit. The characteristic chemical shifts that were observed at 6.88 ppm and 6.77 ppm are allocated to the four vinylic protons (c.f., Figure 1, peak d). Furthermore, the specific peak of triptycene’s sp3 protons was detected at 6.02 ppm (c.f., Figure 1, peak e). On the other hand, the peaks that were observed in the aliphatic region, namely at 2.79 ppm, 1.49 ppm, 1.39 ppm, and 0.88 ppm, are referred to the butyl side chains. The ^1^H-NMR spectra of **TCP2** and **3** and **OTCP1–3** reveal all of the chemical shifts proving their synthesis (Figures S1–S9 in the Supplementary Materials).

Figure 2 divulges the comparative FTIR absorption spectra of both the thioether-containing copolymer **TCP1** and its derivative **OTCP1** with sulfone units: the asymmetric and symmetric stretching vibration peaks of the sulfone moiety (O=S=O) in **OTCP1** were detected at 1314 cm^−1^ and 1120 cm^−1^, respectively, which strongly confirms the selective oxidation of the thioether units in **TCP1** to their corresponding sulfonated groups in **OTCP1** [18]. Furthermore, the stretching vibration bands of the aromatic =C-H groups of **TCP1** and **OTCP1** were observed at 3055 cm^−1^, whereas the stretching vibration peaks of their aliphatic –C–H groups were noticed at 2958 cm^−1^ [81]. Similarly, the absorption peak that was detected at 1670 cm^−1^ is referred to C=N groups stretching vibrations, whereas the one which appears at 1592 cm^−1^ is assigned to the aromatic C=C stretching vibrations. In addition, the bands that were observed at 1464 cm^−1^ and 818 cm^−1^ are ascribed to the aliphatic C-H groups bending vibrations, whereas the C=C bending vibrations of conjugated alkenes were detected at 701 cm^−1^. It is noteworthy that the target copolymers **TCP2 and 3** and their derivatives **OTCP2 and 3** also revealed distinctive peaks which signifies the success of their syntheses (Figures S22–S24 in the Supplementary Materials) [82,83,84].

The thermal stability of **TCP1–3** was investigated by the means of thermogravimetric analysis (TGA) which portrayed a 10% weight loss temperature ranging from 136 °C to 271 °C. Nevertheless, the oxidation reaction of the hitherto mentioned copolymers into **OTCP1–3**, which bear stiffer poly(vinyl sulfone) units, led to relatively higher thermal stability thermograms as evidenced by their 10% weight loss temperatures the ranges of which were found between 269 °C and 299 °C (Figure 3). 

The elemental composition of **copolymers TCP1–3** and **OTCP1–3** was explored by the use of X-ray photoelectron spectroscopy (XPS), the survey scan spectra of which corroborated the presence of all of the elemental peaks (Figures S25–S30 in the Supplementary Materials). Figure 4 portrays the XPS spectrum of **TCP2**, which confirms the existence of all of the constituents, i.e., carbon, oxygen, nitrogen, boron, sulfur, and iron [85]. The C1s peak of **TCP2** can be fitted into two main binding energy values at ~284.67 eV and 285.36 eV, where the former corresponds to the aromatic carbon groups (C=C) and the latter relates to that of imine carbons (C=N). The binding energy that was detected at ~532.42 eV is assigned to oxygen that is bonded to boron and nitrogen. Additionally, the N1s spectrum exhibited two peaks at 399.36 eV and 400.68 eV, which can be correlated to the carbon–nitrogen (C-N) bonds of Tröger’s base and clathrochelate units, respectively. S2p was detected at 163.55 eV, thus denoting the presence of C-S [75]. On the other hand, The B1s spectra was found at 191.27 eV, which clearly divulges the presence of boron oxide (B-O) [86]. Figure 4 also reveals the XPS peak for Fe2p with binding energy values of 709.30 eV and 721.94 eV, which correspond to Fe(II)-N compounds [87]. Similarly, the target copolymers **TCP1 and 3** disclosed conclusive XPS binding energy values, which undoubtedly prove their formation as well (Figures S7–S10 in the Supplementary Materials). It should be mentioned that, unlike **TCP1–3**, copolymers **OTCP1–3** divulged two main binding energy values for oxygen; one at ~532.33 eV (which corresponds to oxygen that is bonded to boron and nitrogen) and a second peak at ~533.30 eV (which is assigned to oxygen that is bonded to sulfur) [88]. Furthermore, the S2p binding energy of copolymers **TCP1–3** that was detected at ~163.50 eV [89] had shifted to 167.90 eV [88], which clearly confirms the complete oxidation of the thioether groups into their corresponding sulfone moieties in **OTCP1–3** (Figures S25–S30 in the Supplementary Materials). 

As can be noticed from Table 1, the good solubility of **TCP1–3** and **OTCP1** in polar organic solvents has allowed for the determination of their molar weights by gel permeation chromatography (GPC), disclosing a weight average molar mass (Mw) ranging from 20 kDa to 30 kDa and a number average molar mass (Mn) values varying between 6.3 kDa and 10 KDa. These results show a polydispersity index (PDI = Mw/Mn) in the range of 2.8 to 3.3 (Table 1 and Figures S31–S34 in the Supplementary Materials). Unfortunately, the scarce solubility of copolymers **OTCP2 and 3** prevented the determination of their GPC chromatograms.

### 3.4. Copolymers’ Adsorption of Lithium Ions

The target copolymers **TCP1–3** and **OTCP1–3** were tested as lithium ion adsorbents by adding 5 mg of a given copolymer sample into a 5 mL aqueous solution of Li**^+^** with an initial concentration 200 mg L^−1^ at ambient temperature, overnight. It is noteworthy that **OTCP2** (i.e., the poly(vinyl sulfone) copolymer bearing Tröger base units) revealed the highest Li**^+^** uptake capacity of 17.88 mg g^−1^, followed by **OTCP3** and **OTCP2** which displayed lithium ions adsorption capacities of 14.46 and 13.22 mg g^−1^, respectively. On the other hand, the poly(vinyl sulfide) compounds revealed lithium ion uptake capacities in the range of 3.93 to 9.12 mg g^−1^ (see Table S1 in the Supplementary Materials). These results suggest that the sulfone units bestow copolymers **OTCP1–3** with a higher polarizability when compared to their poly(vinyl sulfide) synthons **TCP1–3**, thus leading to greater lithium ion uptake that is further improved when the copolymer backbone contains the bowl-shaped Tröger base units (i.e., in the case of **OTCP2)**. Thus, in order to better understand the adsorption affinity towards lithium ions, the adsorption isotherm was studied by adding the latter copolymer into solutions with different initial concentrations of Li**^+^** ranging from 10 to 300 mg L^−1^ at ambient temperature, overnight. The Li**^+^** concentration in the solution was measured by ICP-MS after an adsorption equilibrium was reached and the equilibrium adsorption capacity of **OTCP2** for lithium q_e_ (mg g^−1^) was determined using the following equation:

q_e_·(mg g^−1^) = (C_o_ − C_e_)V/m, (1)

where C_o_ and C_e_ are the initial and equilibrium concentrations of the Li**^+^** solutions (mg L^−1^), respectively, m (g) is the quantity of the adsorbent **OTCP2** that was used, and V (L) is the volume of the Li**^+^** solution [32].

**Figure 5:**
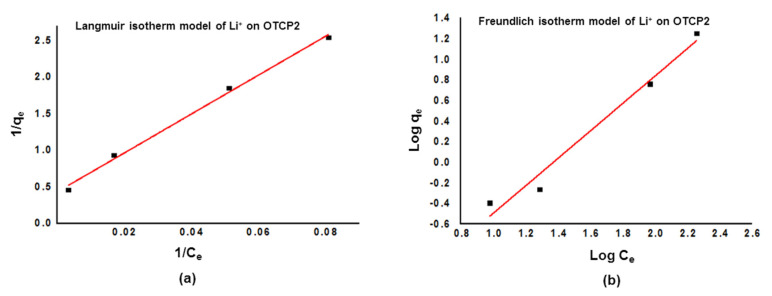
Langmuir isotherm (**a**) and Freundlich isotherm (**b**) models of Li**^+^** on **OTCP2**.

As is shown in Figure 5, two isotherm models (Langmuir and Freundlich) were used to fit the experimental data. As can be noticed from Table 2, the correlation coefficient, R^2^, value of the Langmuir isotherm model was found to be equal to 0.9934, which is significantly higher than the value corresponding to the Freundlich isotherm model (0.9645). This, therefore, suggests that the Langmuir isotherm is more applicable to explaining the equilibrium data for the adsorption of lithium ions on adsorbent **OTCP2**. Accordingly, the maximum adsorption capacity (q_m_) that was computed from the Langmuir model for Li**^+^** was found to be 2.31 mg g^−1^, which is promising when compared to several of the values that have been reported in the literature [90,91,92,93,94,95] (Table S2 in the Supplementary Materials). This is especially so when taking into account the versatile synthesis of the metalorganic copolymers that have been reported herein and the relative ease of making various derivatives from them in order to improve their lithium ion uptake.

### 3.5. Methylene Blue Adsorption Tests

**Figure 6:**
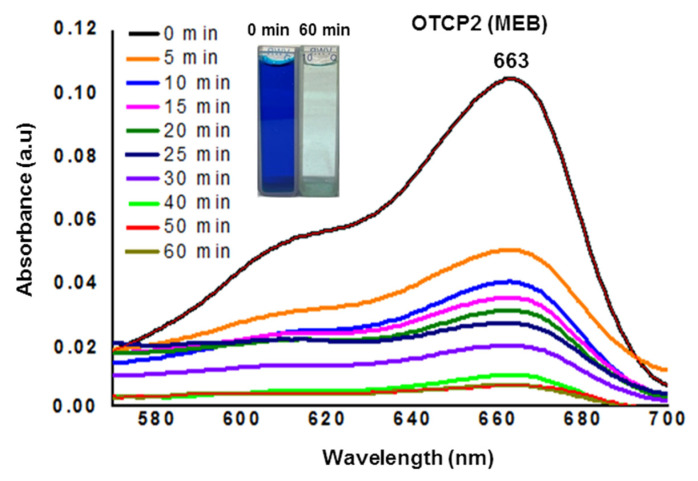
UV–Vis absorption spectrum of MEB aqueous solution (50 mg L^−1^) in the presence of **OTCP2** at various time intervals (inset: samples photos disclosing the color change after dye adsorption).

Given its high Li**^+^** adsorption capacity, **OTCP2’s** uptake performance was tested for another cationic species, methylene blue (MEB) dye, which is believed to be a primary source of water pollution [96,97,98]. **OTCP2’s** capacity to adsorb MEB was studied by stirring 5 mg of the former into a 5 mL aqueous solution of the latter dye (50 mg L^−1^) at ambient temperature and its concentration before and after adsorption was determined by the use of UV/Vis spectrophotometry (Figure 6). Figure 6 reveals that the **OTCP2’s** adsorption capacity for MEB from an aqueous solution attained a level of 87% in 60 min as proven by the sharp reduction in the latter characteristic maximum absorption peak that was detected at ~660 nm. The adsorption efficiency, E (%), and the quantity of MEB that was adsorbed by **OTCP2**, q_e_ (mg g^−1^), were calculated using the following equations:

E (%) = (C_o_ − C_e_) /C_o_ X 100, (2)

q_e_ (mg g^−1^) = (C_o_ − C_e_) V/m (3)

where C_o_ and C_e_ denote the initial and equilibrium MEB concentrations (mg L^−1^), respectively, m (g) is the amount of the adsorbent **OTCP2** that was used, and V (L) is the volume of the MEB solution [75].

Furthermore, in order to explore the maximum adsorption capacity (q_m_) of MEB by **OTCP2**, adsorption isotherms experiments were carried out by preparing a series of aqueous solutions of MEB with initial concentrations ranging from 50 to 600 mg L^−1^. The Langmuir and Freundlich isotherm models were utilized in order to fit the adsorption isotherm data that were determined for MEB. The following linear equation was applied for the Langmuir isotherm model:

1/q_e_ = 1/K_L_q_m_ X 1/C_e_ + 1/q_m_ (4)

The linear equation that was used for the Freundlich isotherm model was expressed as:

Log q_e_ = Log K_F_ + 1/n Log C_e_ (5)

where q_e_ (mg g^−1^) expresses the equilibrium adsorption capacity, C_e_ (mg L^−1^) denotes the dye concentration at equilibrium, and q_m_ (mg g^−1^) is the maximum adsorption capacity. K_L_ is the Langmuir constant whereas K_F_ and n are the Freundlich constants that are correlated to the sorption capacity and sorption intensity, respectively [99]. 

**Figure 7:**
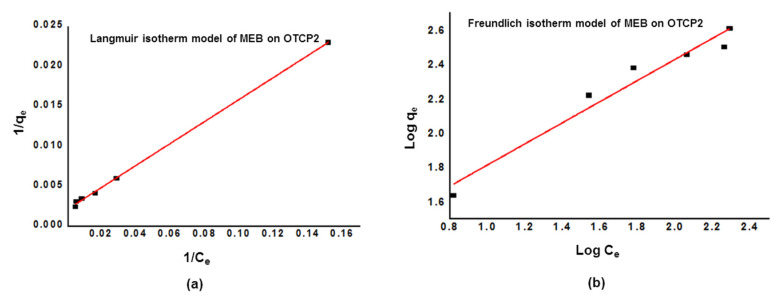
Langmuir isotherm (**a**) and Freundlich isotherm (**b**) models of MEB on **OTCP2**.

Figure 7 portrays the Langmuir plot of 1/q_e_ versus 1/C_e_ whereas the values of the Freundlich isotherm were determined from the plot of log q_e_ versus log C_e_. Both of these graphical representations were employed in order to fit the equilibrium data that were collected regarding MEB adsorption (Table 3). The correlation coefficient, R^2^, that was derived from the linear plot of the Langmuir model (R^2^ = 0.9988) was found to be higher than that which was computed from the Freundlich isotherm model (R^2^ = 0.9480) for the adsorption of MEB (Table 3 and Figure 7). This finding strongly implies that the Langmuir isotherm model is more favorable for its use to describe the equilibrium data, which suggests the homogenous adsorption and formation of an MEB monolayer on the adsorbent **OTCP2**. In addition, the maximum adsorption capacity (q_m_) that was extrapolated from the Langmuir model for MEB was found to be 480.77 mg g^−1^, which is, to the best of our knowledge, higher than the adsorption capacity values for several adsorbents that have been reported in the literature [100,101,102,103,104] (Table S3 in the Supplementary Materials). It is worthwhile to mention that the nonlinear fittings [105] of the parameters of both the Freundlich and Langmuir isotherm models did not afford better correlation coefficients (see Figure S36 in the Supplementary Materials).

The adsorption mechanism of MEB on **OTCP2** was investigated by carrying out kinetic experiments using an initial MEB concentration of 50 mg L^−1^ at different time intervals. As can be noticed from Figure 6, the copolymer’s adsorption capacity for MEB increases sharply within the first 15 min and reached equilibrium in 60 min. Thus, to better understand the adsorption kinetics, pseudo first-order and pseudo second-order kinetic models were employed.

The pseudo first-order model is expressed by the following equation:

ln(q_e_ − q_t_) = ln q_e_ − k_1_t (6)

The linear equation that is detailed below was employed in order to investigate the pseudo second-order model:

t/q_t_ = t/q_e_ + 1/k_2_q_e_^2^ (7)

where q_e_ (mg g^−1^) and q_t_ (mg g^−1^) are the adsorption capacities at equilibrium and time t (min), respectively. The variable k_1_ is the rate constant of the pseudo first-order model, whereas k_2_ is the rate constant of the pseudo second-order model [99]. 

**Figure 8:**
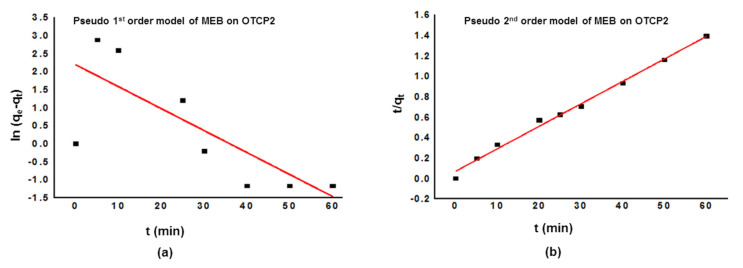
Pseudo first-order (**a**) and pseudo second-order (**b**) models of MEB on **OTCP2**.

As is shown in Figure 8, the calculated adsorption capacity at equilibrium, q_e,cal_, was obtained from the pseudo first-order model by plotting ln(q_e_ – q_t_) versus t, whereas a plot of t/q_t_ versus t was employed in order to determine q_e,cal_ from the pseudo second-order model. Interestingly, the data that were compiled for both of the models in Table 4, below, reveal that the correlation coefficient, R^2^, that was derived from the linear correlation using the pseudo second-order model (R^2^ = 0.9919) is higher than the one that was derived from the pseudo first-order model (R^2^ = 0.4983). Moreover, Table 4 discloses the values of the experimental and calculated capacities at equilibrium, q_e_,_exp_ and q_e,cal_, respectively, revealing a better agreement between the former value with that of the calculated capacity at equilibrium that was derived from the pseudo second-order model. This strongly suggests that that the adsorption of MEB by **OTCP2** follows the pseudo second-order kinetic model wherein both the adsorbate and adsorbent contribute to the reaction and which rather follows the chemisorption mechanism [106].

Regeneration experiments were carried out in order to test the adsorbing performance of **OTCP2** towards MEB after several adsorption–desorption cycles wherein the former was loaded with MEB and ultrasonicated in deionized water for 10 min. This was followed by its isolation by vacuum filtration over a membrane filter and this procedure was repeated for three successive times in order to ensure that **OTCP2** was fully regenerated before adding it to a freshly prepared solution of MEB, as is described in Section 3.5. These tests were repeated for several cycles and they ultimately rendered a removal efficiency of 96%, even after three cycles (c.f. Figure S38 in the Supplementary Materials).

## 4. Conclusions

In summary, three highly soluble copolymers that are composed of iron(II) clathrochelate with lateral butyl chains and intercalated by various contorted units containing the thioether groups **TCP1–3** were synthesized in a one-step reaction. Subsequently, the copolymers underwent selective oxidation of their thioether units into their corresponding sulfone derivatives, affording copolymers **OTCP1–2** in excellent yields. Lithium ion adsorption tests of the target copolymers revealed the maximum adsorption capacity by **OCTP2** of 2.31 mg g^−1^. Furthermore, **OTCP2** disclosed excellent adsorption capacity for the cationic dye methylene blue (MEB) from an aqueous solution, it exhibited a maximum adsorption capacity (q_m_) of 480.77 mg g^−1^ (by following the Langmuir isotherm model). The kinetic study of MEB adsorption by **OTCP2** suggested a pseudo second-order model disclosing an equilibrium adsorption capacity, q_e,cal_, of 45.40 mg g^−1^. Thus, the copolymers that are presented herein disclose several advantages in addition to their versatile and environmentally friendly synthesis, including their cost-effectiveness and superior stability. They have also been revealed to be promising adsorbents of lithium ions and methylene blue which qualify them as prominent adsorbents for water purification applications. 

## Figures and Tables

**Table 1:** Summary of the GPC results of the copolymers **TCP1–3** and **OTCP1**.

Entry	Polymer	M_w_ (g·mol^−1^)	M_n_ (g·mol^−1^)	Đ
1	TCP1	30198	10066	3.0
2	TCP2	21404	7493	2.8
3	TCP3	21061	6315	3.3
4	OTCP1	20017	7056	2.8

**Table 2:** Langmuir and Freundlich isotherm model parameters for Li**^+^** adsorption on **OTCP2**.

Ion on OTCP2	Langmuir Isotherm Parameters			Freundlich Isotherm Parameters		
	q_m_ (mg g^−1^)	K_L_	R^2^	1/n	K_F_	R^2^
Li^+^	2.31	0.01691	0.9934	1.3302	0.1605	0.9645

**Table 3:** Langmuir and Freundlich isotherm model parameters for MEB adsorption on **OTCP2**.

Dye on OTCP2	Langmuir Isotherm Parameters			Freundlich Isotherm Parameters		
	q_m_ (mg g^−1^)	K_L_	R^2^	1/n	K_F_	R^2^
**MEB**	480.77	0.01508	0.9988	0.6136	3.3211	0.9480

**Table 4:** Pseudo first-order and pseudo second-order kinetic model parameters for the adsorption MEB on **OTCP2**.

Dye on OTCP2			Pseudo 1st Order Model			Pseudo 2nd Order Model		
	C_o_ (mg L^−1^)	q_e,exp_ (mg g^−1^)	q_e,cal_ (mg g^−1^)	k_1_ (min^−1^)	R^2^	q_e,cal_ (mg g^−1^)	k_2_ (min^−1^)	R^2^
**MEB**	50	43.41	8.88	−0.001	0.4983	45.40	0.0069	0.9919

## Data Availability

The raw/processed data that were required to reproduce these findings can be shared upon demand.

## Supplementary Materials

The following supporting information can be downloaded at: https://www.mdpi.com/article/10.3390/polym14163394/s1, Figures S1–S9: ^1^H-NMR spectra of TC1-3, TCP1-3 and OTCP1-3; Figures S10–S18: ^13^C-NMR spectra of TC1-3, TCP1-3 and OTCP1-3; Figures S19–S21: EI-HRMS spectra of TC1-3; Figures S22–S24: Comparative FTIR spectra of TCP1-3 and OTCP1-3; Figures S25–S30: High-resolution XPS spectra of TCP1-3 and OTCP1-3; Figures S31–S34: Normalized GPC chromatogram of TCP1-3, OTCP1; Table S1: Summary of the Li+ adsorption by TCP1-3, OTCP1-3; Table S2: Comparison table for Li+ adsorption capacity of OTCP2 and various adsorbents published in the literature; Table S3: Comparison table for MEB adsorption capacity of OTCP2 and various adsorbents published in the literature; Figure S35: Nonlinear Langmuir and Freundlich isotherm models of Li+ on OTCP2; Figure S36: Nonlinear Langmuir and Freundlich isotherm models of MEB on OTCP2; Figure S37: Nonlinear Pseudo first- and second-order models of MEB on OTCP2; Figure S38: Regeneration tests results of OTCP2 to adsorb MEB.
