# Supplementary material for: Synthesis of Metalorganic Copolymers Containing Various Contorted Units and Iron(II) Clathrochelates with Lateral Butyl Chains: Conspicuous Adsorbents of Lithium Ions and Methylene Blue
Source: Polymers (Basel). 2022 Aug 19;14(16):3394. doi: 10.3390/polym14163394 (PMC9412635; doi:10.3390/polym14163394)
Supplement: Supplementary file 1 [file polymers-14-03394-s001.zip › polymers-1883481-supplementary.pdf]

Supporting Information

Synthesis of Metalorganic Copolymers Containing Various Contorted Units and Iron (II) Clathrochelates with Lateral Butyl Chains: Conspicuous Adsorbents of Lithium Ions and Methylene Blue

Suchetha Shetty,[1,2] *Noorullah Baig,*[1,2] *Moustafa Sherief Moustafa* [3]*, Saleh Al-Mousawi,*[3] *and Bassam Alameddine**[1,2]

[1,2] Suchetha Shetty, Noorullah Baig, and Bassam Alameddine

[1]Department of Mathematics and Natural Sciences, Gulf University for Science and Technology, Kuwait.

[2]Functional Materials Group – GUST, Kuwait.

E-mail: alameddine.b@gust.edu.kw

[3] Moustafa Sherief Moustafa and Saleh Al-Mousawi,

[3]Department of Chemistry, Kuwait University, Kuwait.

Contents

**^{1}H-NMR spectra of TC1-3, TCP1-3 and OTCP1-3**	**Figure S1-S9**
**^{13}C-NMR spectra of TC1-3, TCP1-3 and OTCP1-3**	**Figure S10-S18**
**EI-HRMS spectra of TC1-3**	**Figure S19-S21**
**Comparative FTIR spectra of TCP1-3 and OTCP1-3**	**Figure S22-S24**
**High-resolution XPS spectra of TCP1-3 and OTCP1-3**	**Figure S25-S30**
**Normalized GPC chromatogram of TCP1-3, OTCP1**	**Figure S31-S34**
**Summary of the Li^{+} adsorption by TCP1-3, OTCP1-3**	**Table S1**
**Comparison table for Li^{+} adsorption capacity of OTCP2 and various adsorbents published in the literature**	**Table S2**
**Comparison table for MEB adsorption capacity of OTCP2 and various adsorbents published in the literature**	**Table S3**
**Nonlinear Langmuir and Freundlich isotherm models**	

of Li^{+} on OTCP2 | Figure S35
Nonlinear Langmuir and Freundlich isotherm models
of MEB on OTCP2 | Figure S36
Nonlinear Pseudo first- and second-order models
of MEB on OTCP2 | Figure S37
Regeneration tests results of OTCP2 to adsorb MEB | Figure S38

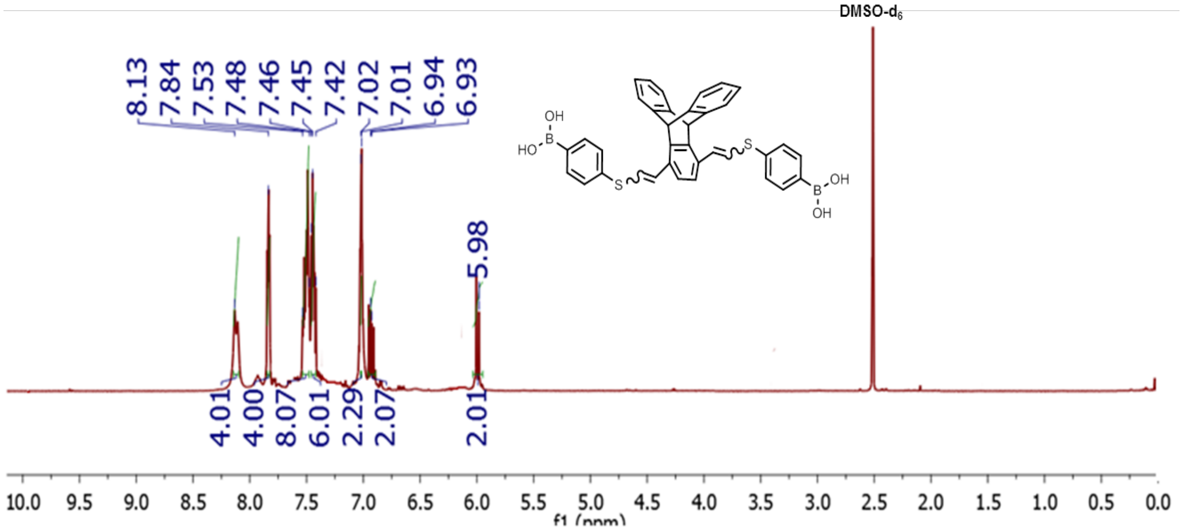

Figure S1 ^{1}H NMR spectrum of **TC1**

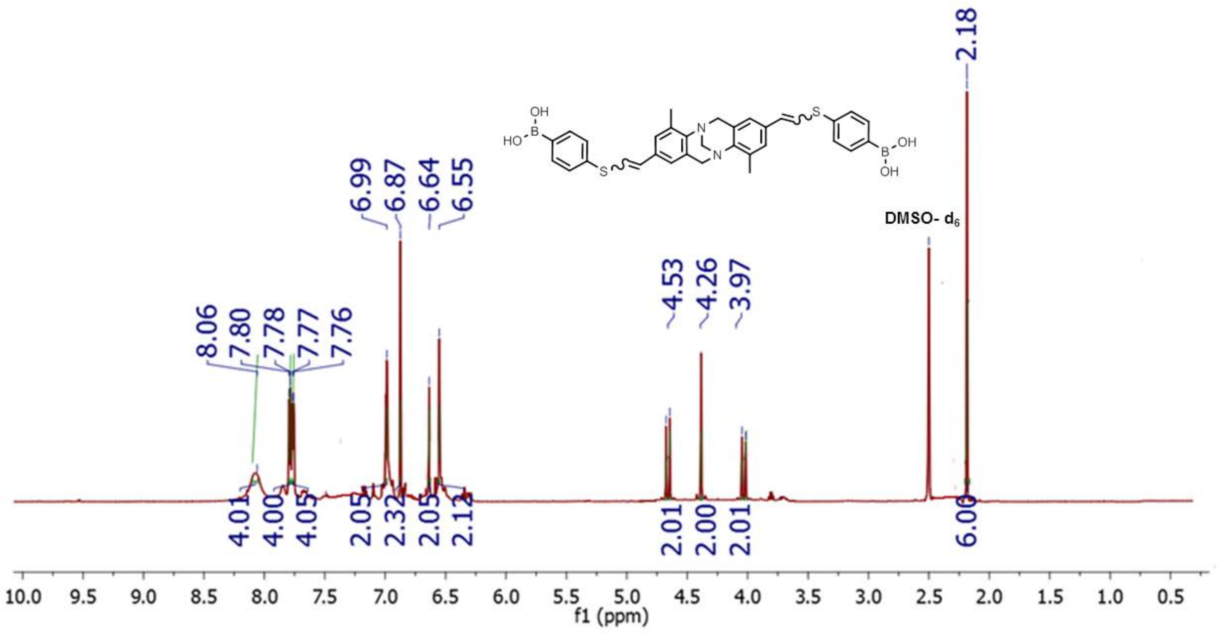

Figure S2 ^{1}H NMR spectrum of **TC2**

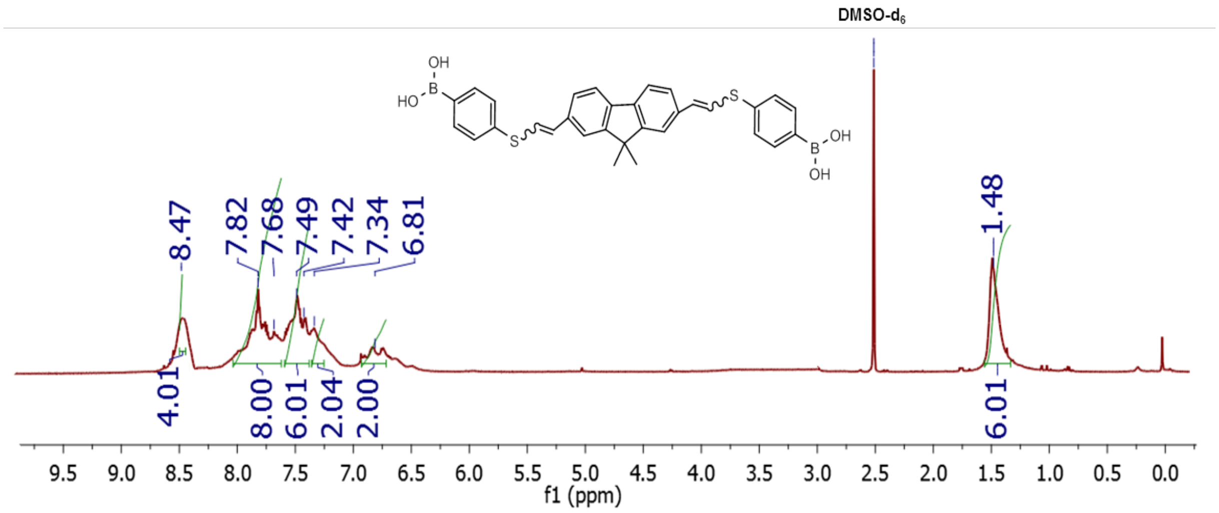

Figure S3 ^{1}H NMR spectrum of **TC3**

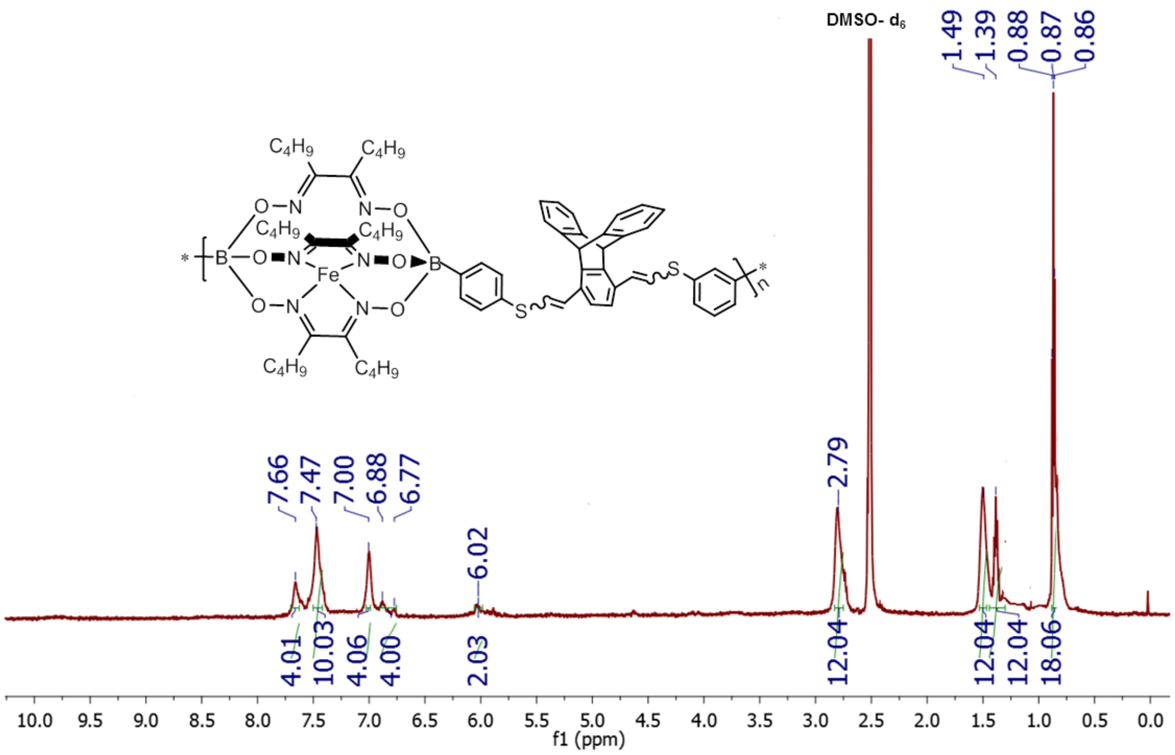

Figure S4 ^{1}H NMR spectrum of **TCP1**

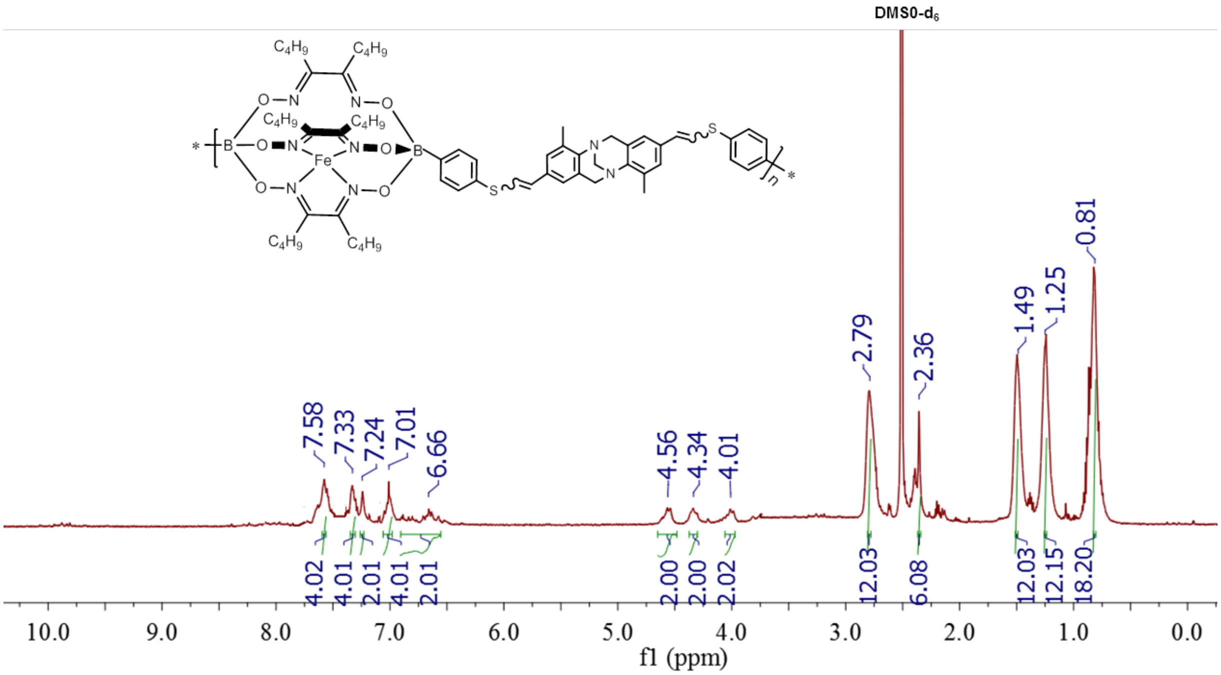

Figure S5 ^{1}H NMR spectrum of **TCP2**

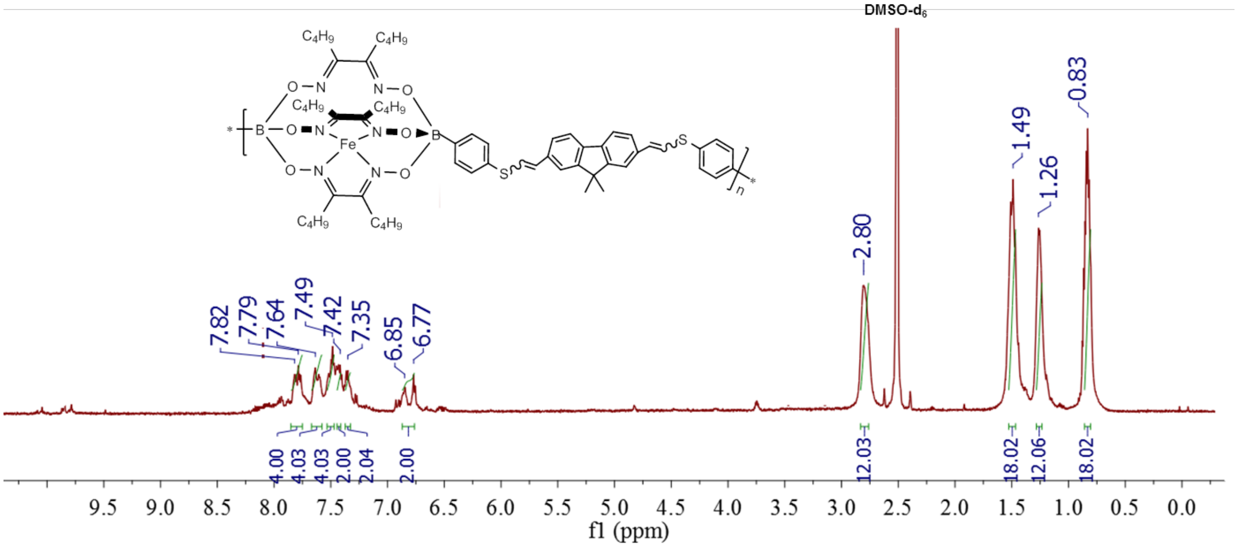

Figure S6 ^{1}H NMR spectrum of **TCP3**

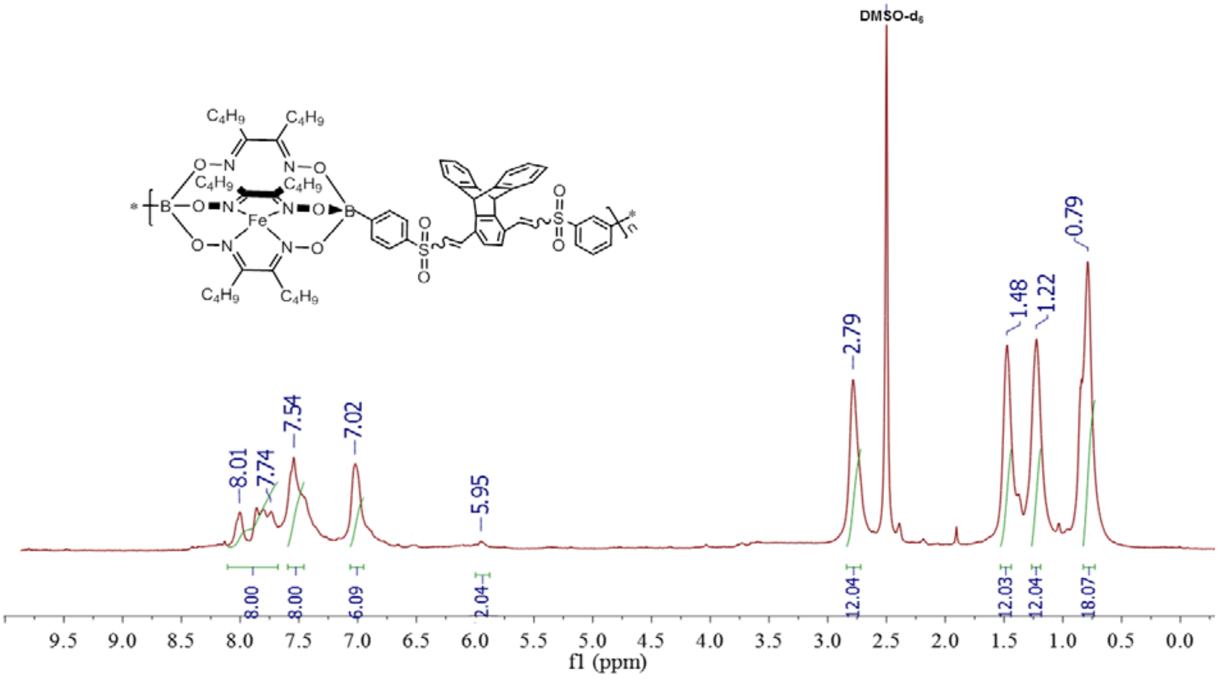

Figure S7 ^{1}H NMR spectrum of **OTCP1**

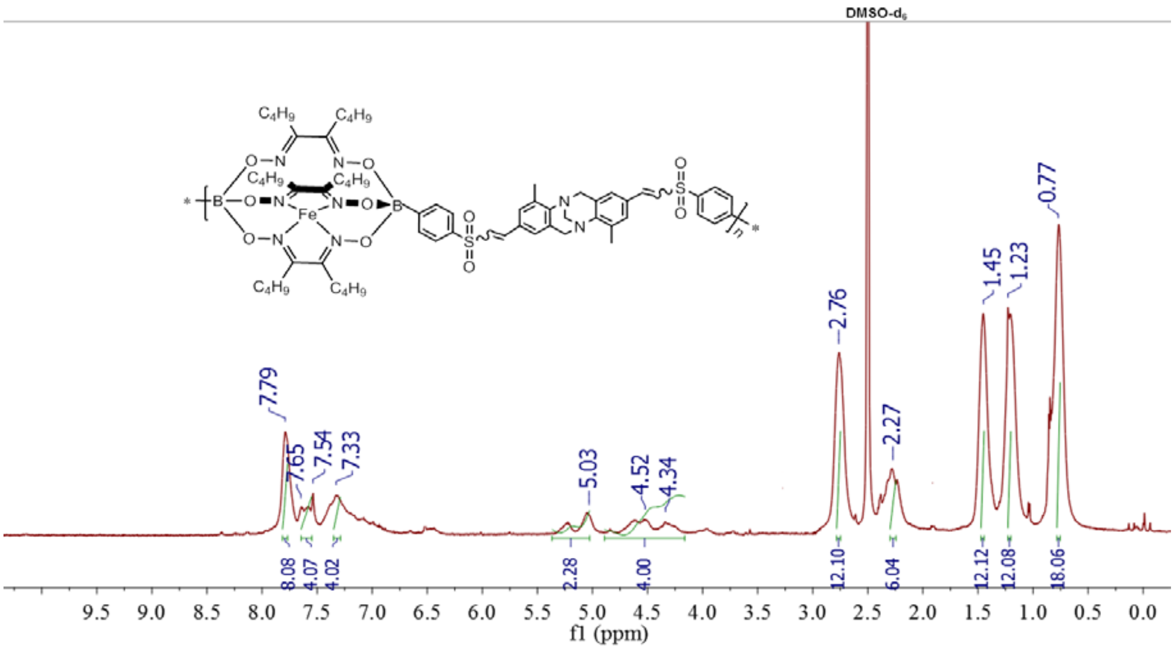

Figure S8 ^{1}H NMR spectrum of **OTCP2**

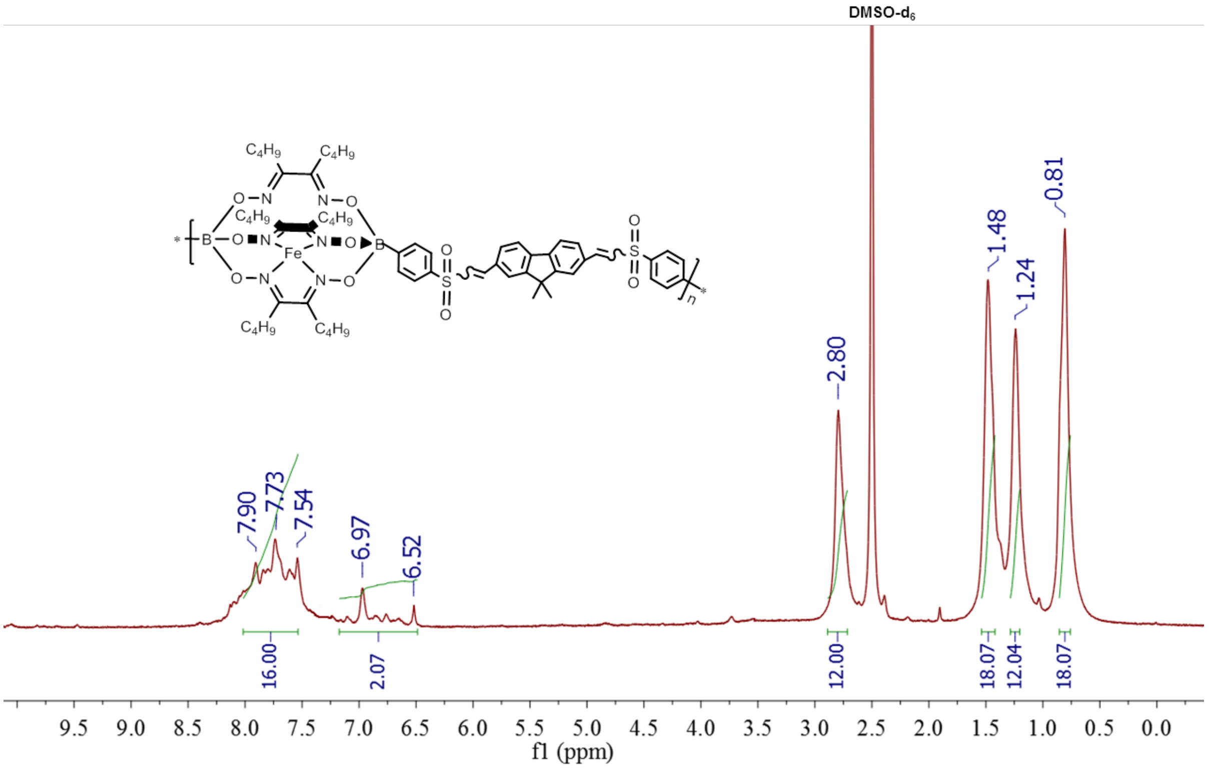

Figure S9 ^{1}H NMR spectrum of **OTCP3**

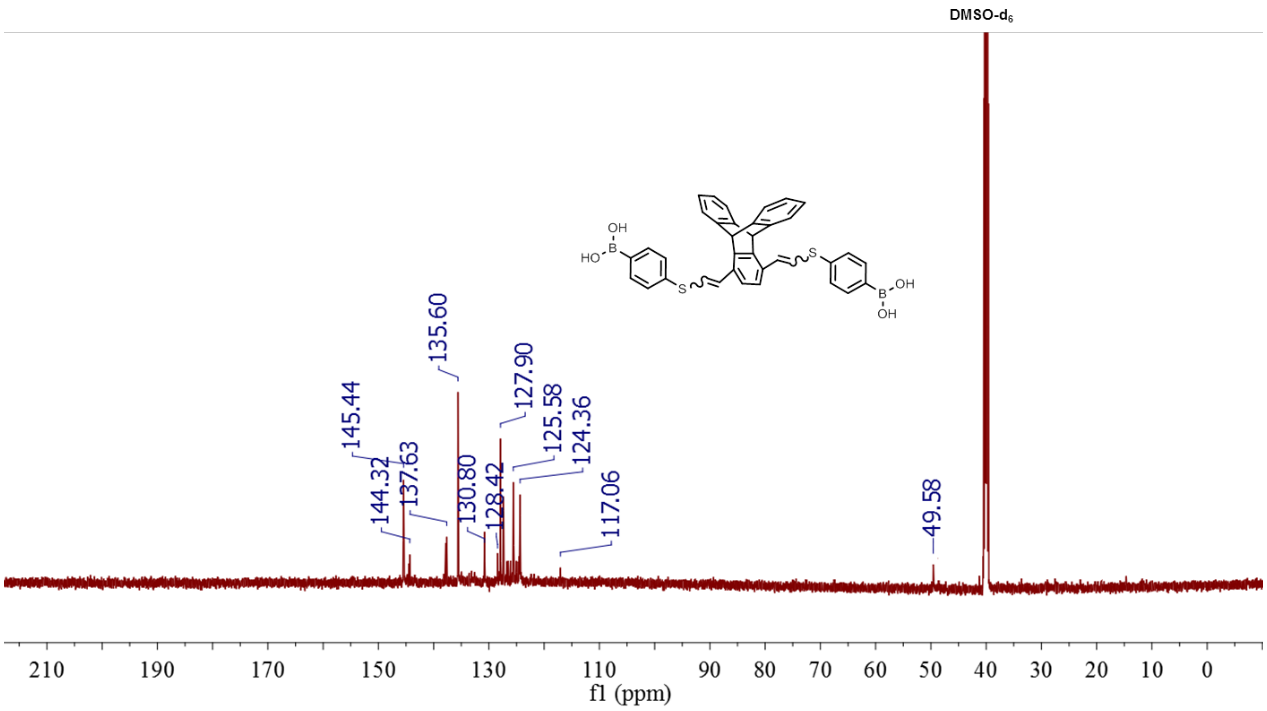

Figure S10 ^{13}C NMR spectrum of **TC1**

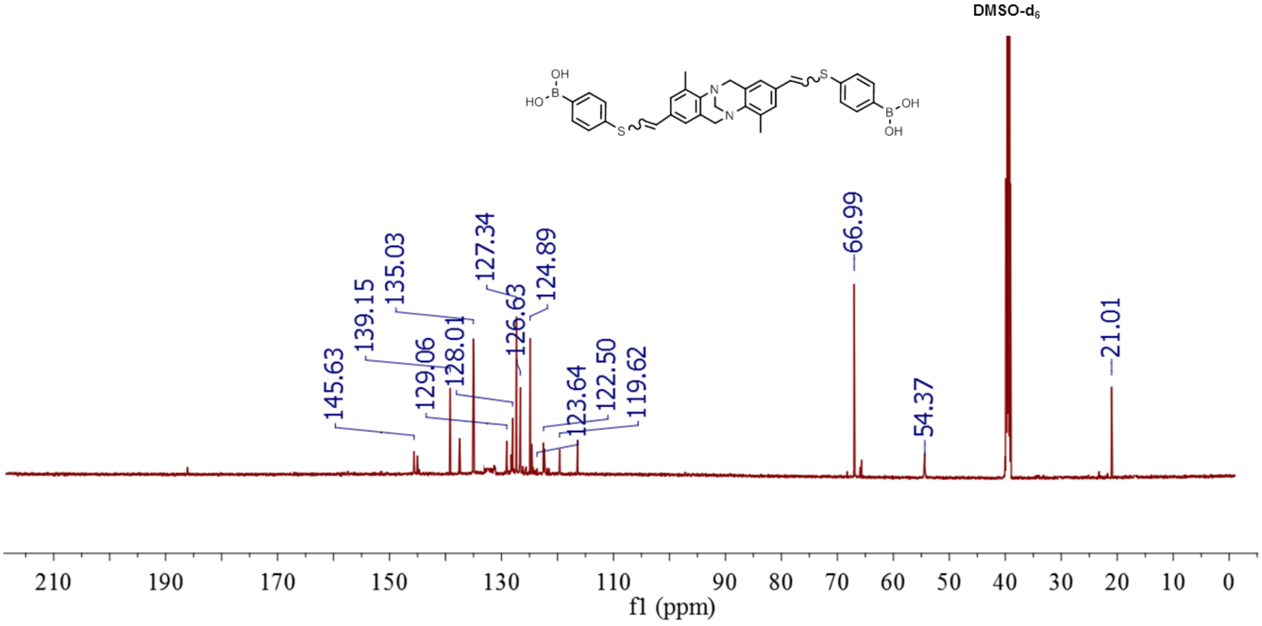

Figure S11 ^{13}C NMR spectrum of **TC2**

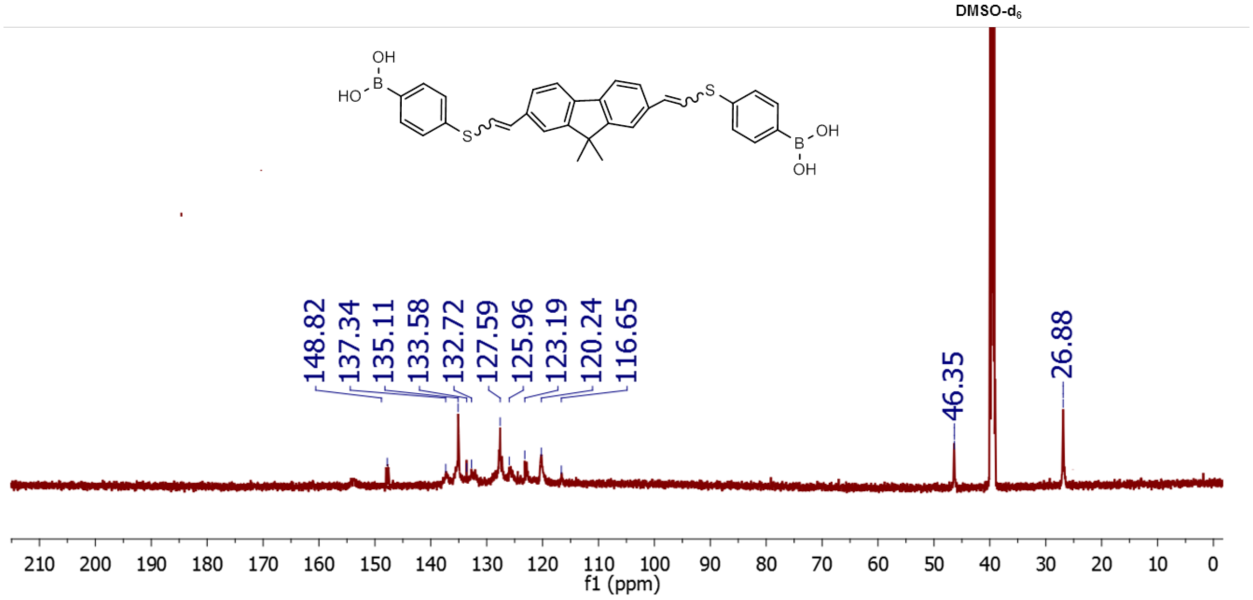

Figure S12 ^{13}C NMR spectrum of **TC3**

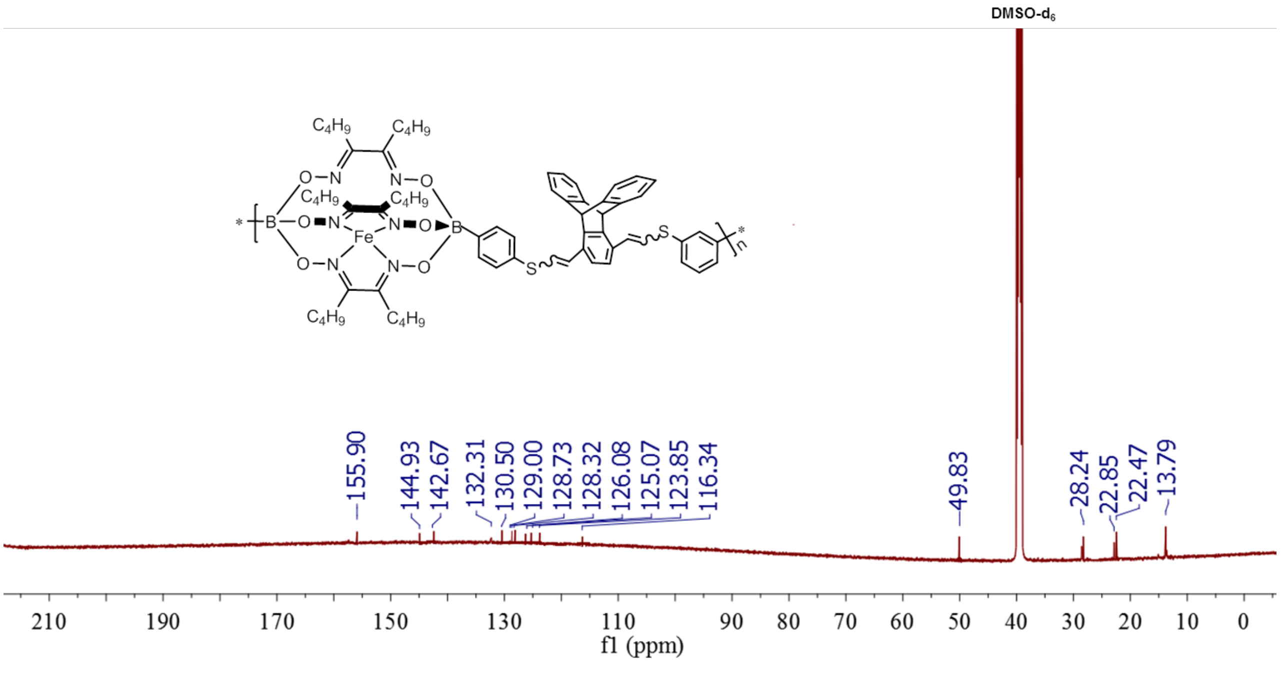

Figure S13 ^{13}C NMR spectrum of **TCP1**

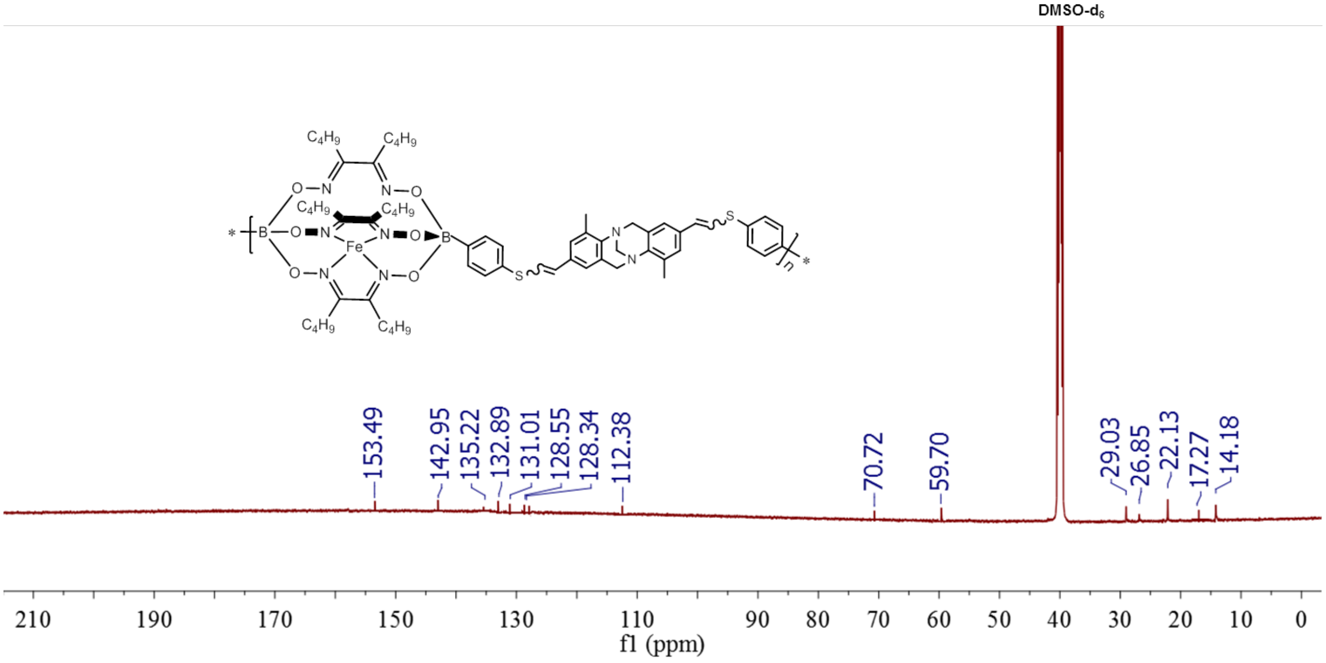

Figure S14 ^{13}C NMR spectrum of **TCP2**

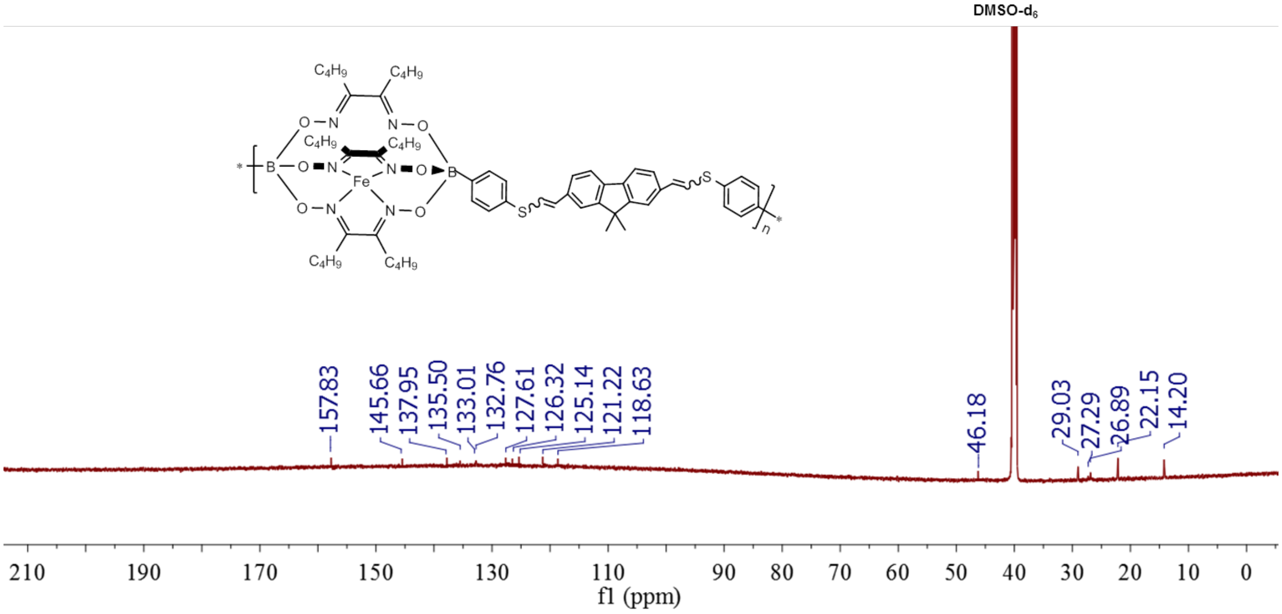

Figure S15 ^{13}C NMR spectrum of **TCP3**

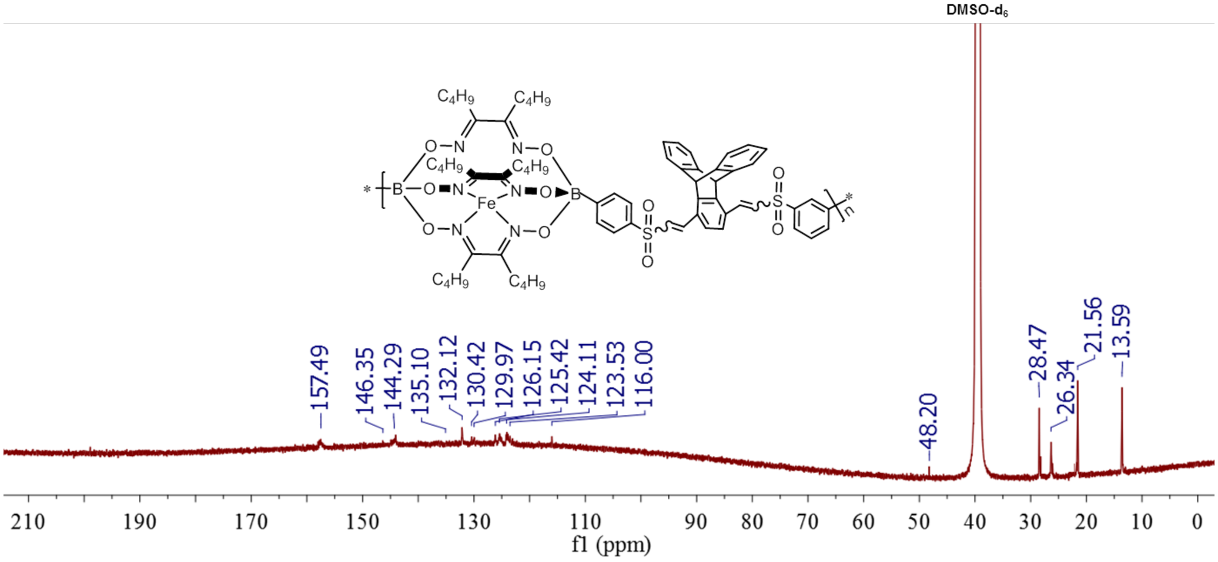

Figure S16 ^{13}C NMR spectrum of **OTCP1**

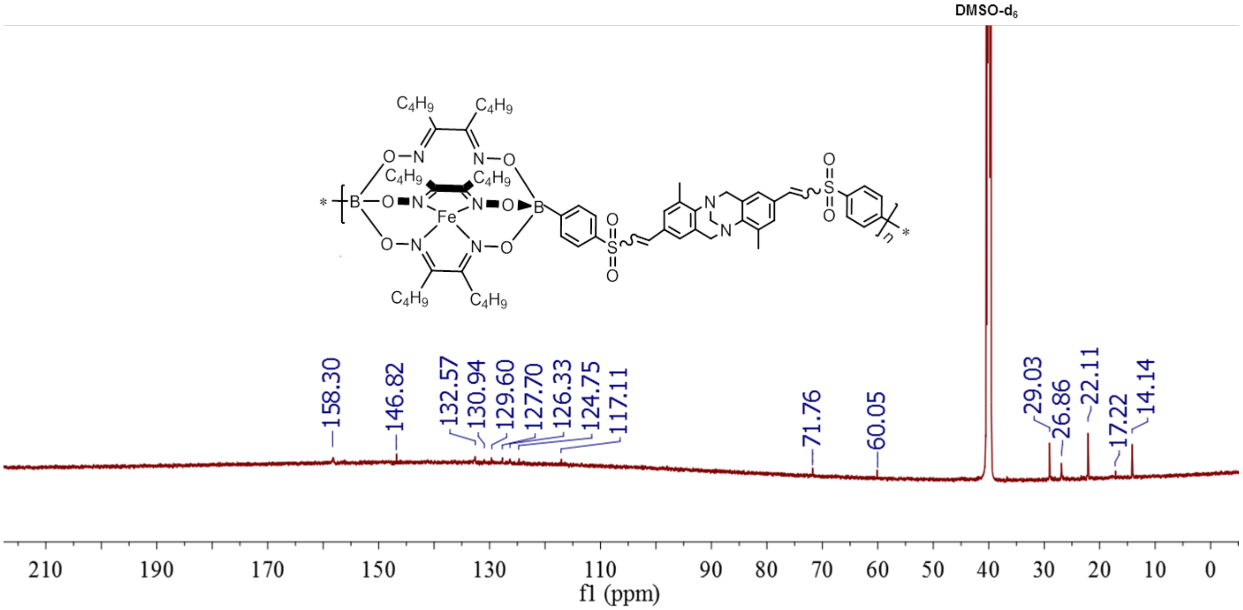

Figure S17 ^{13}C NMR spectrum of **OTCP2**

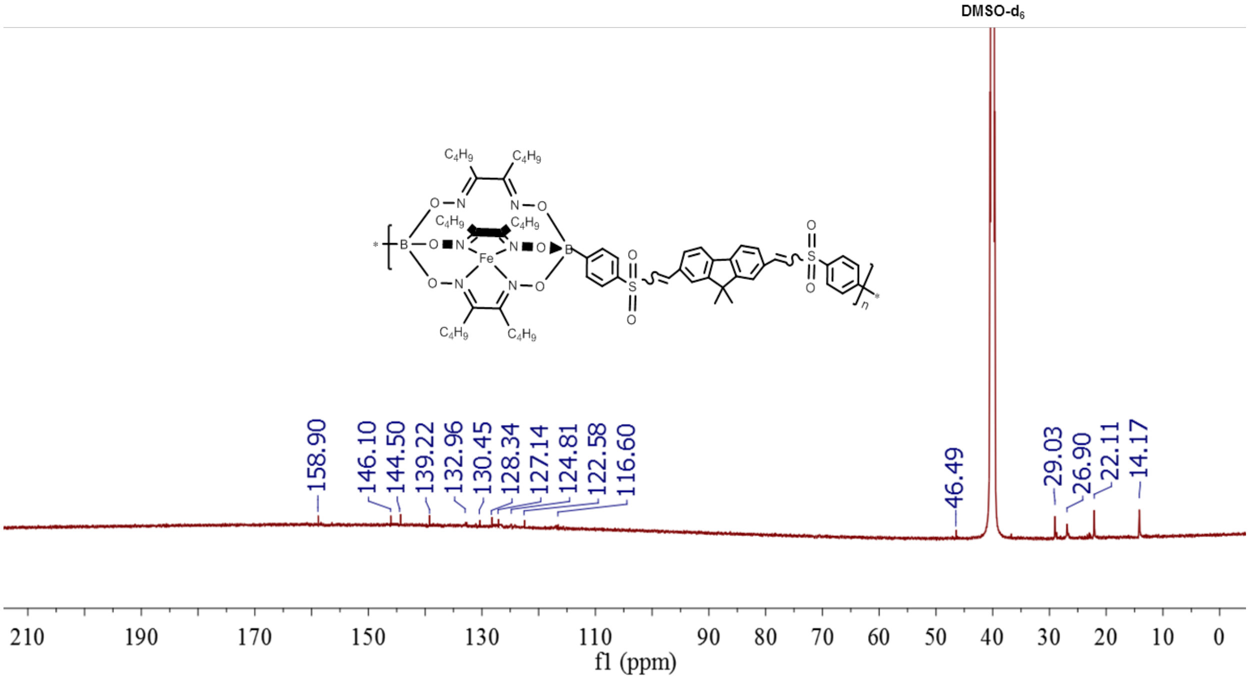

Figure S18 ^{13}C NMR spectrum of **OTCP3**

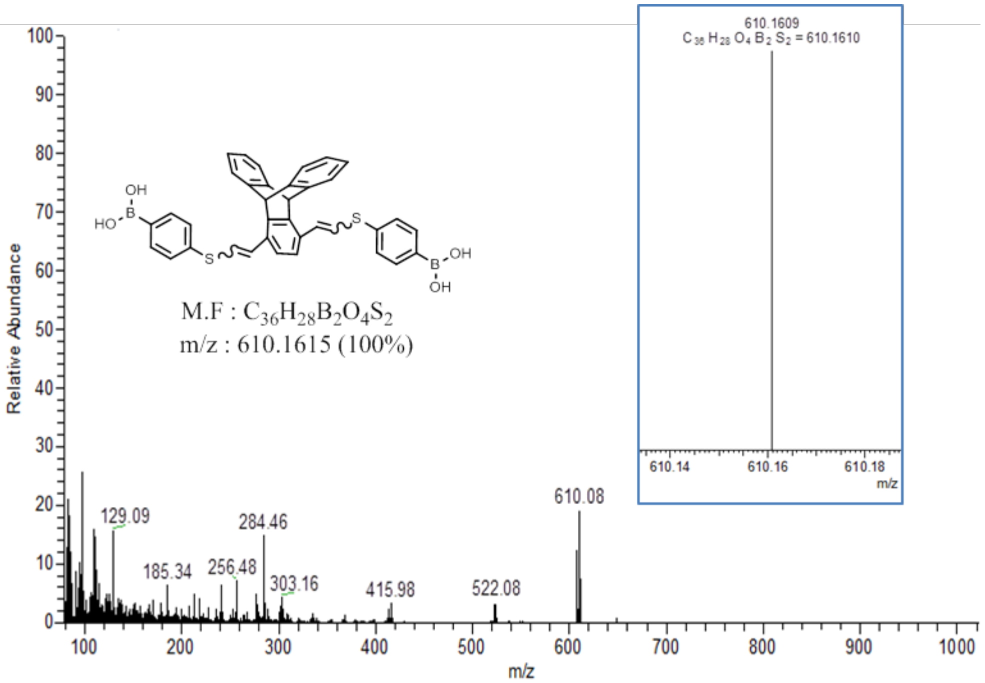

Figure S19 EI-HRMS spectrum of **TC1**

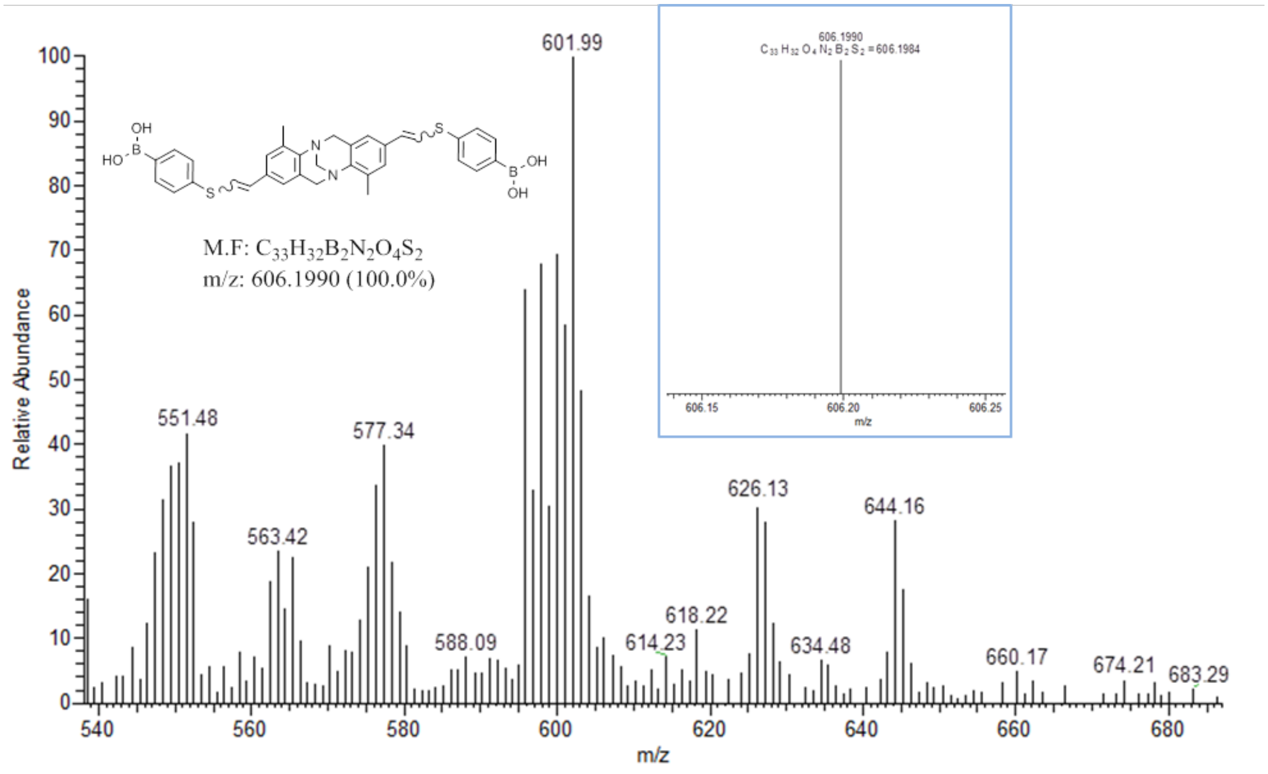

Figure S20 EI-HRMS spectrum of **TC2**

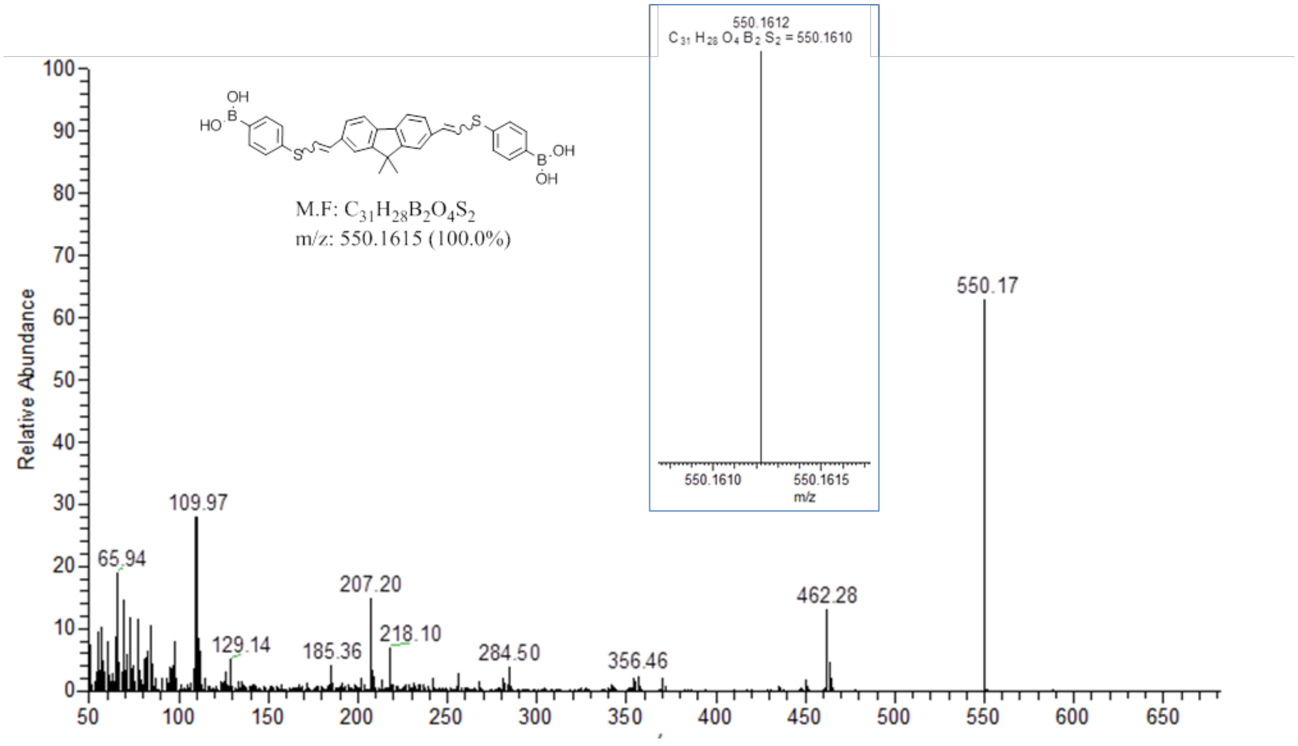

Figure S21 EI-HRMS spectrum of **TC3**

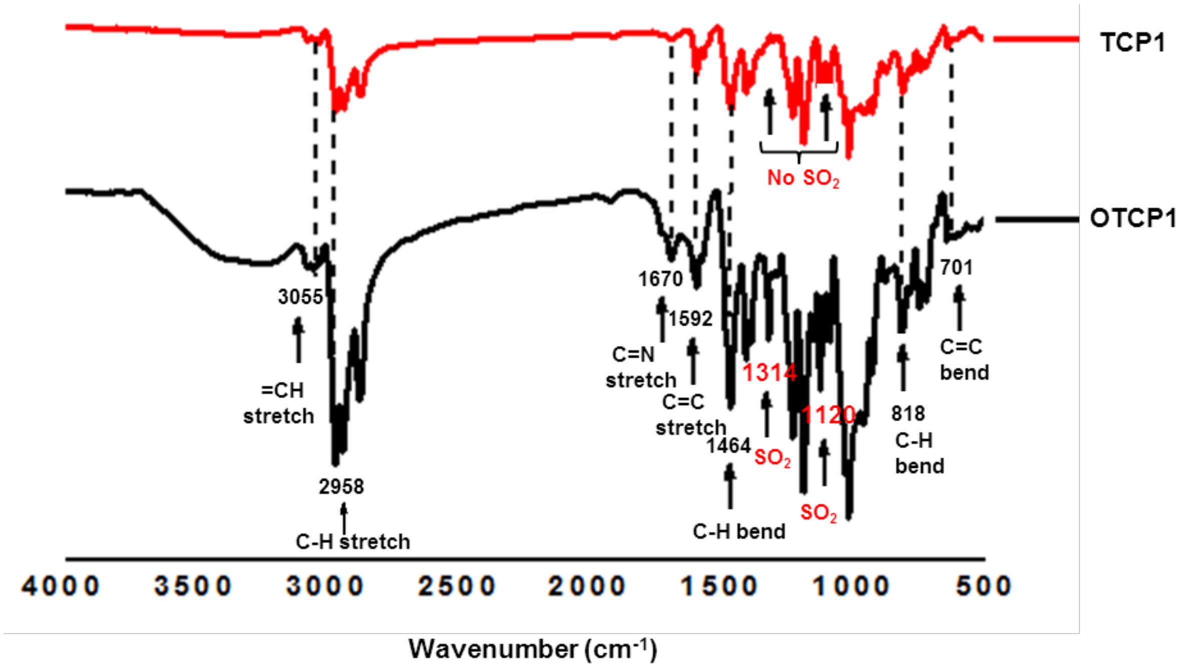

Figure S22 Comparative FTIR spectrum of **TCP1** (up) and **OTCP1** (down)

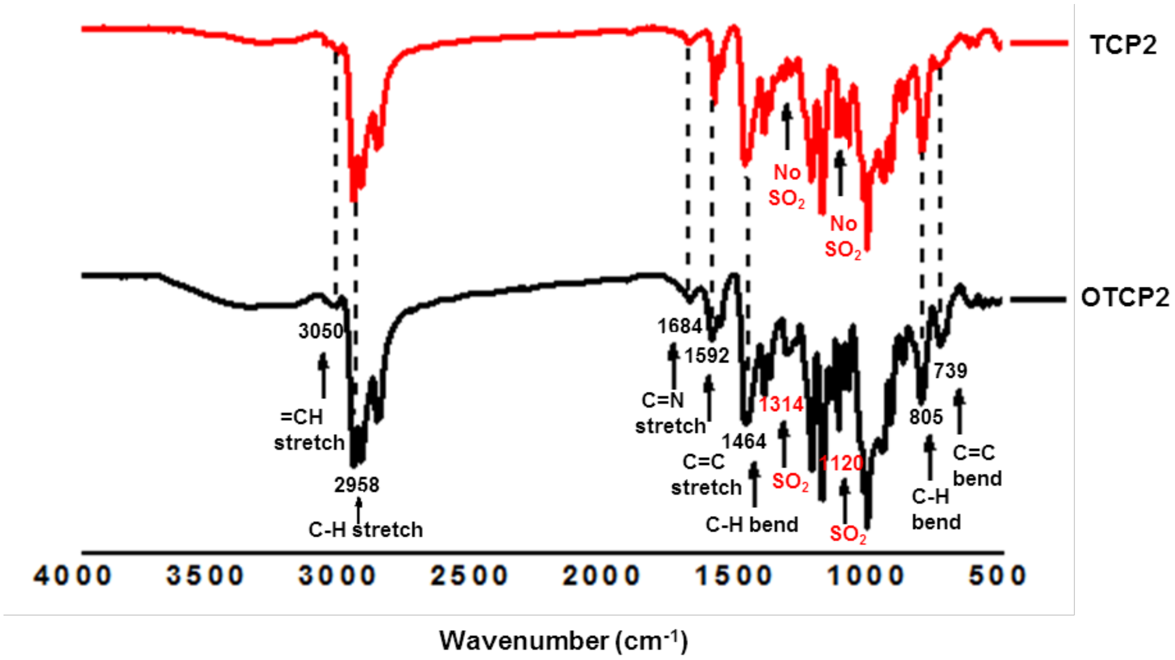

Figure S23 Comparative FTIR spectrum of **TCP2** (up) and **OTCP2** (down)

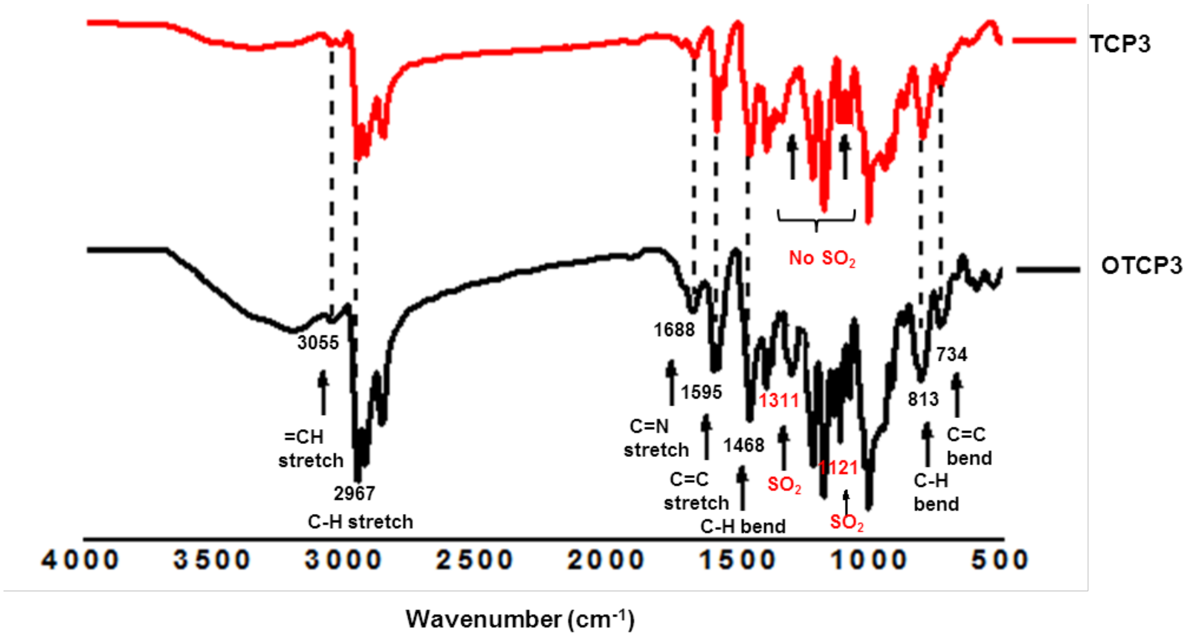

Figure S24 Comparative FTIR spectrum of **TCP3** (up) and **OTCP3** (down)

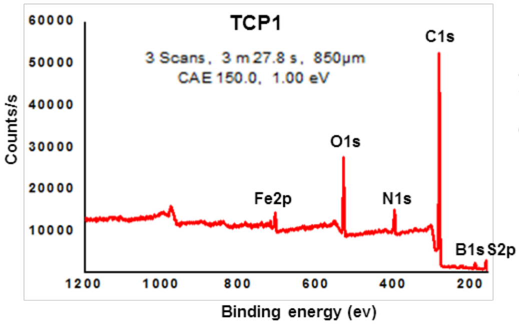

Name	Peak BE
C1s	284.66
C1s A	285.31
O1s	532.49
Fe2p, 3/2	709.23
Fe2p, 1/2	721.98
N1s	400.66
S2p	163.54
B1s	191.25

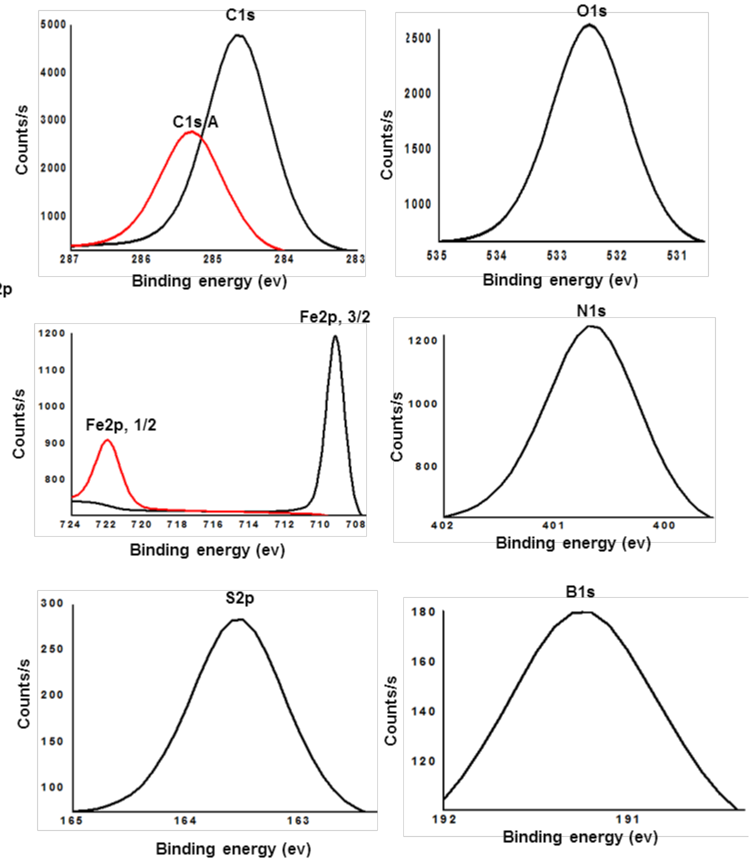

Figure 25 High-resolution XPS spectra of C1s, O1s, Fe2p, N1s, S2p and B1s of **TCP1**

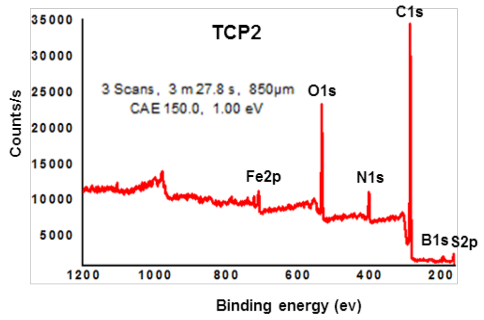

Name	Peak BE
C1s	284.67
C1s A	285.36
O1s	532.42
N1s	400.68
N1s A	399.36
S2p	163.55
Fe2p, 3/2	709.30
Fe2p, 1/2	721.94
B1s	191.27

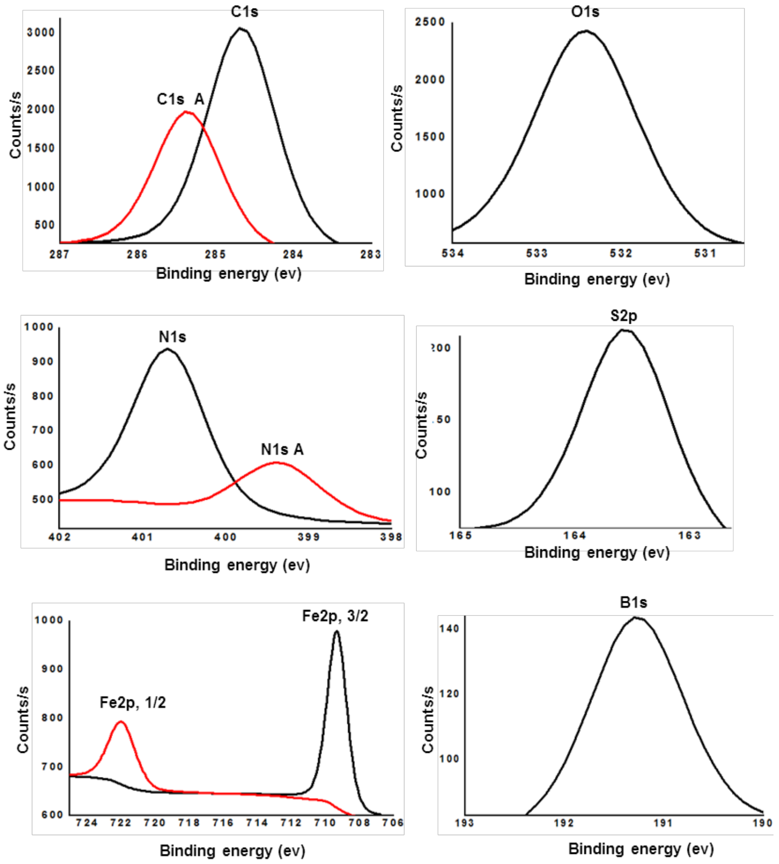

Figure S26 High-resolution XPS spectra of C1s, O1s, N1s, S2p, Fe2p and B1s of **TCP2**

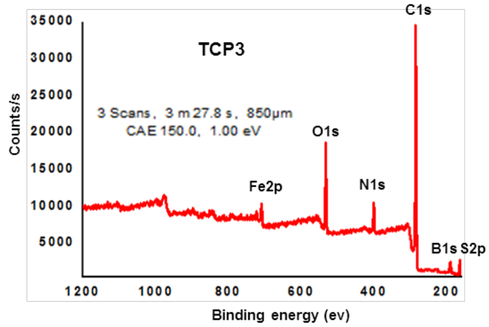

Name	Peak BE
C1s	284.67
C1s A	285.33
O1s	532.64
N1s	400.70
S2p	163.60
Fe2p, 3/2	709.31
Fe2p, 1/2	721.98
B1s	191.22

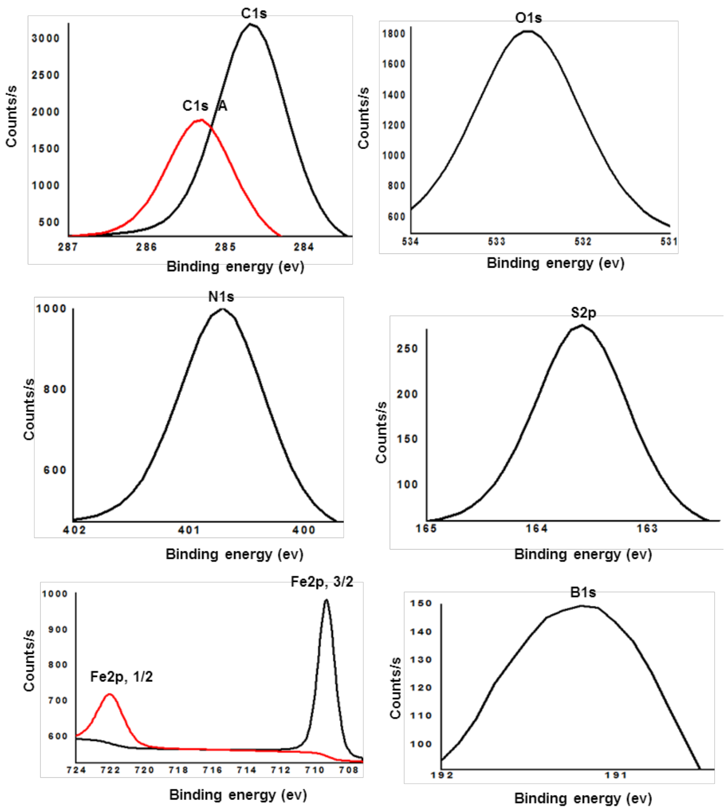

Figure S27 High-resolution XPS spectra of C1s, O1s, N1s, S2p, Fe2p and B1s of **TCP3**

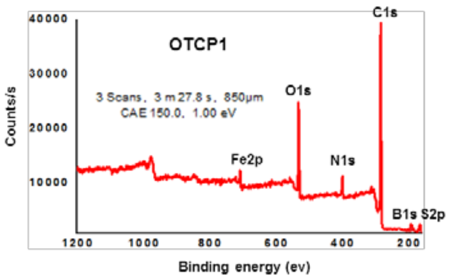

Name	Peak BE
C1s	284.64
C1s A	285.29
O1s	532.26
O1s A	533.22
N1s	400.61
S2p	167.72
Fe2p, 3/2	709.25
Fe2p, 1/2	721.84
B1s	191.19

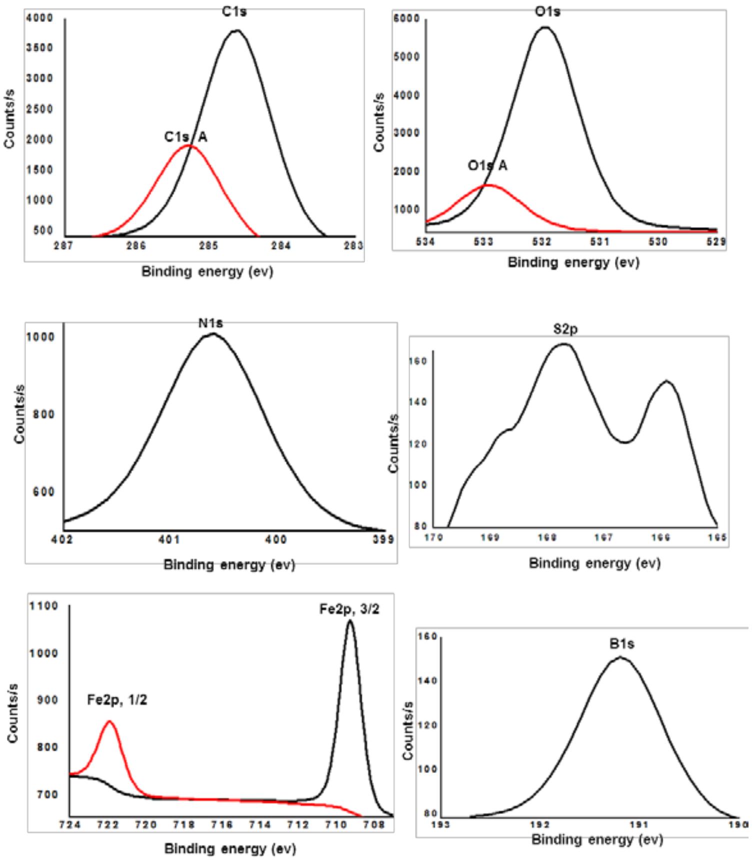

Figure S28 High-resolution XPS spectra of C1s, O1s, N1s, S2p, Fe2p and B1s of **OTCP1**

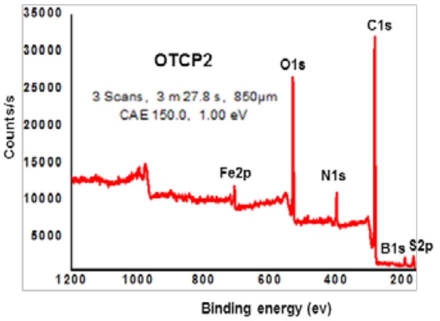

Name	Peak BE
C1s	284.69
C1s A	285.38
O1s	532.33
O1s A	533.30
N1s	400.66
N1s A	399.56
S2p	167.90
Fe2p, 3/2	709.33
Fe2p, 1/2	721.94
B1s	191.32

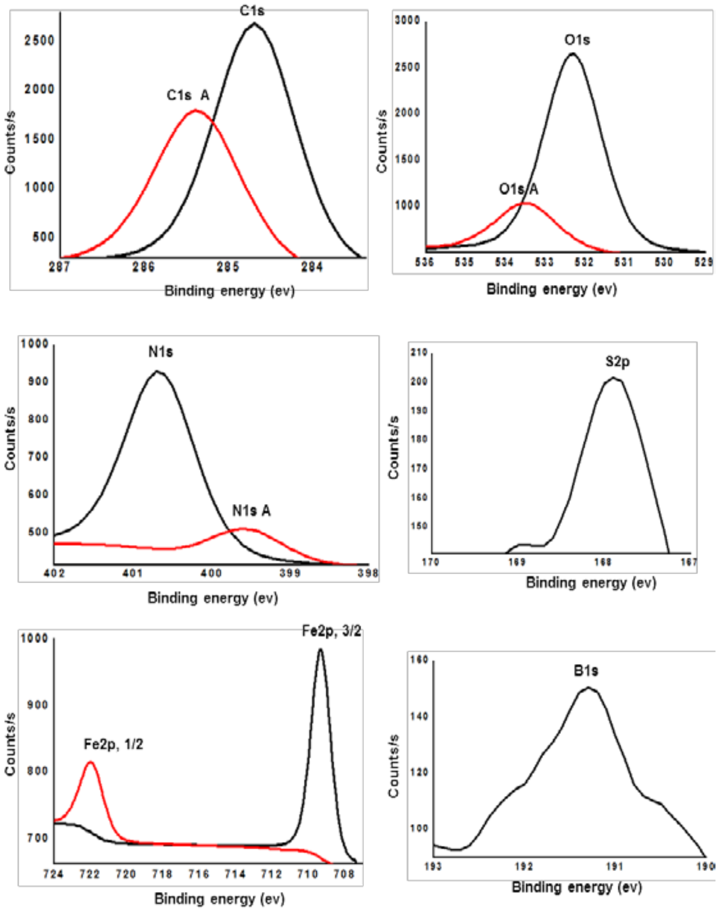

Figure S29 High-resolution XPS spectra of C1s, O1s, N1s, S2p, Fe2p and B1s of **OTCP2**

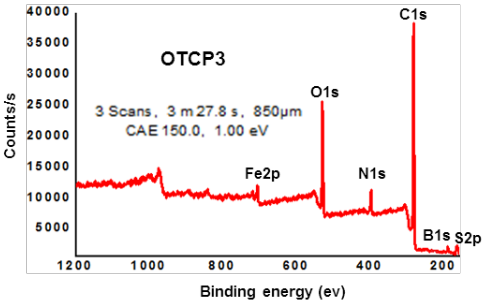

Name	Peak BE
C1s	284.69
C1s A	285.35
O1s	532.54
O1s A	533.53
N1s	400.69
S2p	167.89
Fe2p, 3/2	709.33
Fe2p, 1/2	721.93
B1s	191.21

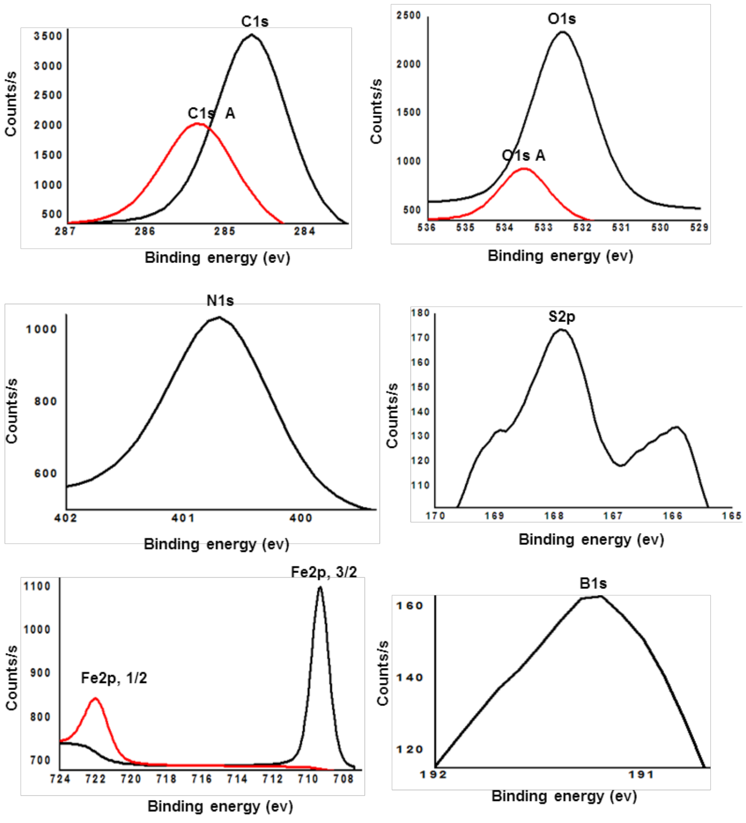

Figure S30 High-resolution XPS spectra of C1s, O1s, N1s, S2p, Fe2p and B1s of **OTCP3**

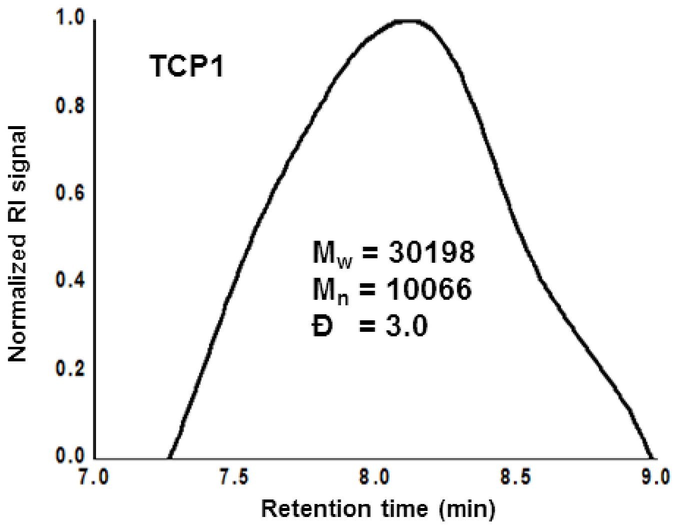

Figure S31 Normalized GPC chromatogram of **TCP1**

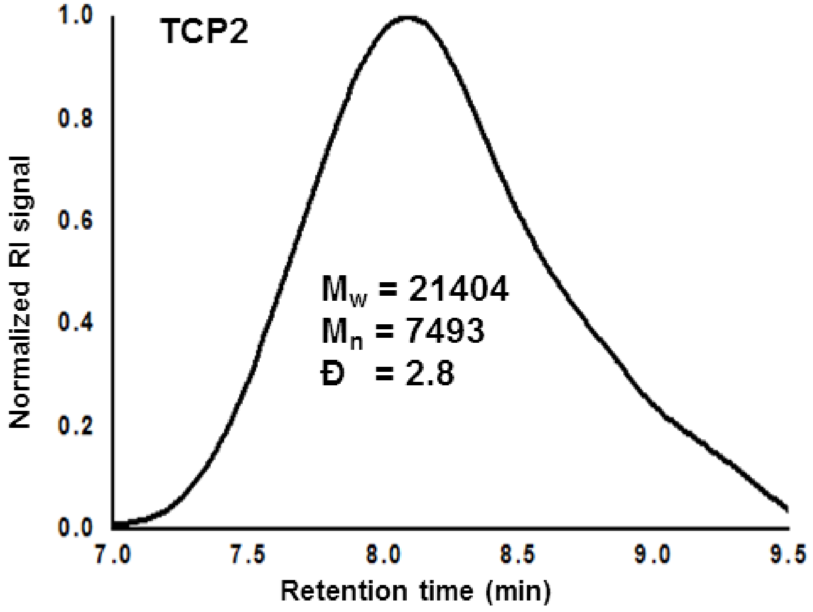

Figure S32 Normalized GPC chromatogram of **TCP2**

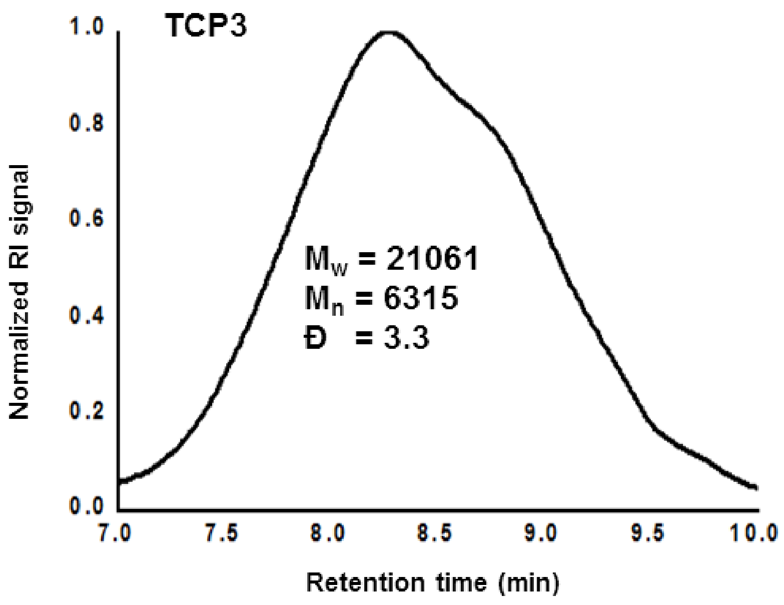

Figure S33 Normalized GPC chromatogram of **TCP3**

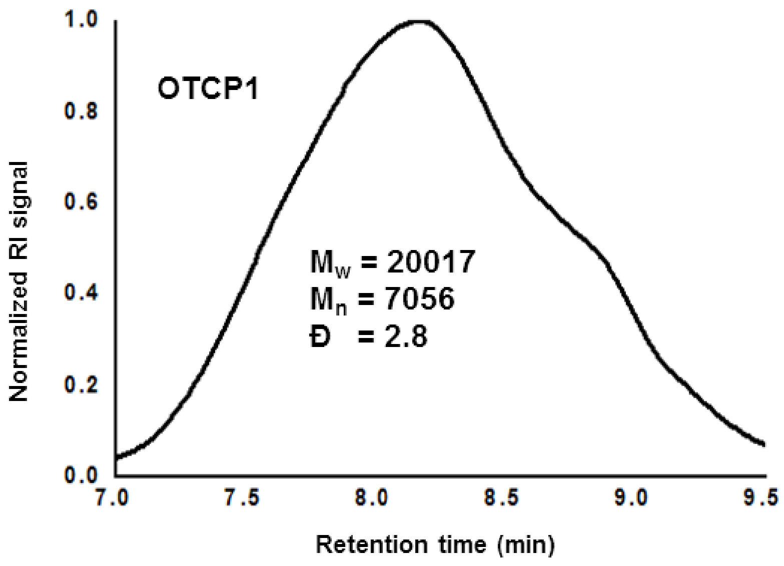

Figures S34 Normalized GPC chromatogram of **OTCP1**

Table S1 Summary of the Li^+ (200 mg L^{-1}) adsorption by copolymers **TCP1-3**, **OTCP1-3**

Entry	Polymer	C_o (mg L^{-1})	Ce (mg L^{-1})	q_e (mg g^{-1})
1	**TCP1**	200	196.07	3.93
2	**TCP2**	200	190.88	9.12
3	**TCP3**	200	194.89	5.11
4	**OTCP1**	200	186.78	13.22
5	**OTCP2**	200	182.12	17.88
6	**OTCP3**	200	185.54	14.46

Table S2 Comparison of adsorption capacity of **OTCP2** with published adsorbents for Li^+

Entry	Adsorbents	Adsorption Capacity (mg g^{-1})	Reference
1	Li/Rb-IHPS	0.166	[1]
2	MWCNT–HDB14C4–COOH	2.11	[2]
3	SG/GO composite	1.1	[3]
4	**OTCP2**	2.31	This work

Table S3 Comparison of adsorption capacity of **OTCP2** with published adsorbents for MEB

Entry	Adsorbents	Adsorption Capacity (mg g^{-1})	Reference
1	ZIF-67-0.2 HPW	446	[4]
2	CS/GO aerogel	437	[5]
3	Graphene aerogel materials	221.77	[6]
4	Composite graphene oxide/nanocellulose aerogel	111.2	[7]
5	**OTCP2**	480.77	This work

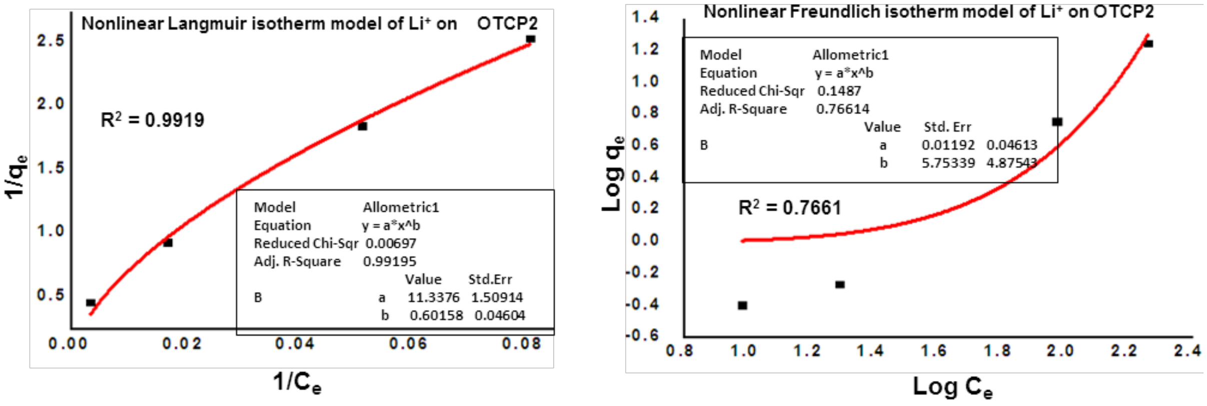

(a) (b)

Figure S35 Nonlinear Langmuir isotherm **(a)** and nonlinear Freundlich isotherm **(b)** models of Li^+ on **OTCP2**

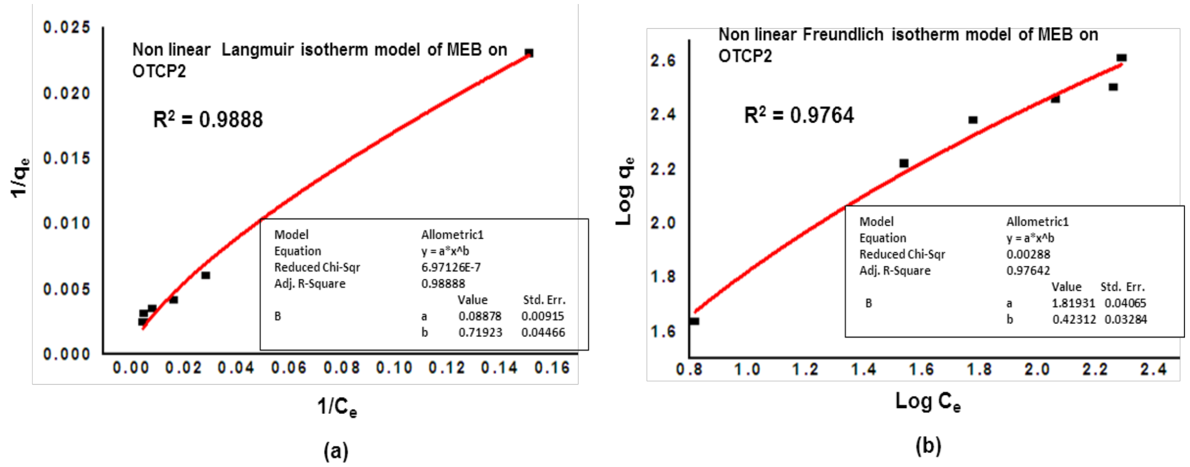

Figure S36 Nonlinear Langmuir isotherm **(a)** and nonlinear Freundlich isotherm **(b)** models of MEB on **OTCP2**

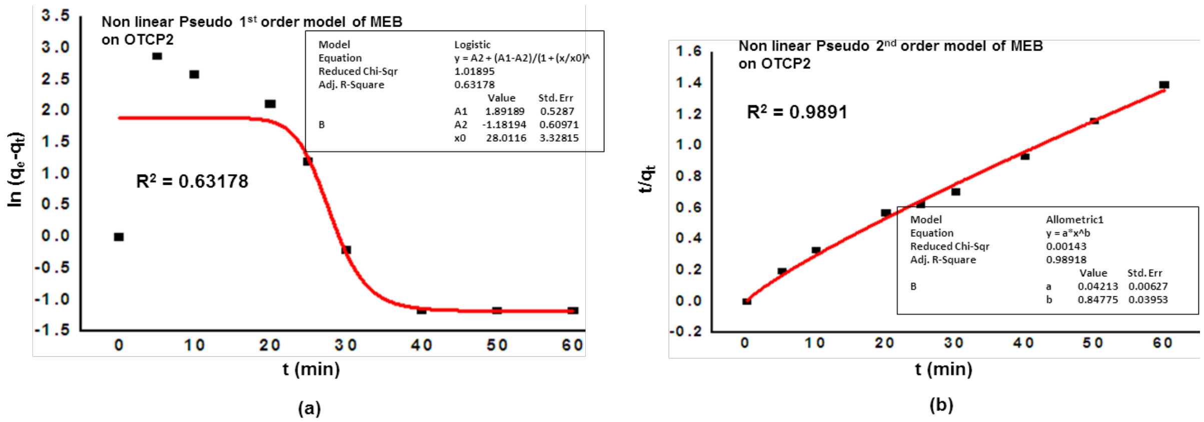

Figure S37 Nonlinear Pseudo first-order **(a)** and nonlinear Pseudo second-order **(b)** models of MEB on **OTCP2**

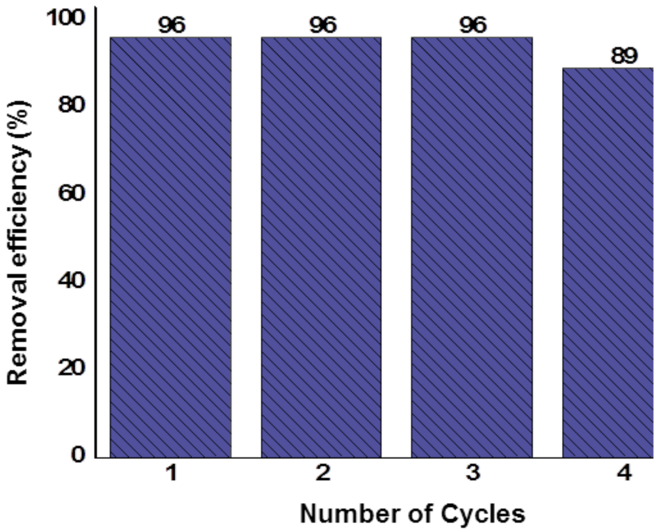

Figure S38 Graphical representation of regeneration capacity of **OTCP2** for the adsorption of methylene blue

References

1. Bai, H.; Xue, C.; Lyu, J.L.; Li, J.; Chen, G.X.; Yu, J.H.; Lin, C.T.; Lv, D.J.; Xiong, L.M. Thermal conductivity and mechanical properties of flake graphite/copper composite with a boron carbide-boron nano-layer on graphite surface. *Composites Part A: Applied Science and Manufacturing* **2018**, *106*, 42-51, doi:https://doi.org/10.1016/j.compositesa.2017.11.019.
2. Torrejos, R.E.C.; Nisola, G.M.; Park, M.J.; Shon, H.K.; Seo, J.G.; Koo, S.; Chung, W.-J. Synthesis and characterization of multi-walled carbon nanotubes-supported dibenzo-14-crown-4 ether with proton ionizable carboxyl sidearm as Li+ adsorbents. *Chemical Engineering Journal* **2015**, *264*, 89-98, doi:https://doi.org/10.1016/j.cej.2014.11.036.
3. Ding, T.; Wu, Q.; Nie, Z.; Zheng, M.; Wang, Y.; Yang, D. Selective recovery of lithium resources in salt lakes by polyacrylonitrile/ion-imprinted polymer: Synthesis, testing, and computation. *Polymer Testing* **2022**, *113*, 107647, doi:https://doi.org/10.1016/j.polymertesting.2022.107647.
4. Wei, S.; Wu, J.; Chen, P.; Fu, B.; Zhu, X.; Chen, M. Integration of Phosphotungstic Acid into Zeolitic Imidazole Framework-67 for Efficient Methylene Blue Adsorption. *ACS Omega* **2022**, *7*, 9900-9908, doi:10.1021/acsomega.2c00377.
5. Xu, W.; Li, Y.; Wang, H.; Du, Q.; Li, M.; Sun, Y.; Cui, M.; Li, L. Study on the Adsorption Performance of Casein/Graphene Oxide Aerogel for Methylene Blue. *ACS Omega* **2021**, *6*, 29243-29253, doi:10.1021/acsomega.1c04938.
6. Trinh, T.; Phuong, T.; Xuan, N.; Nguyet, D.M.; Quan, T.H.; Anh, T.N.M.; Thinh, D.B.; Tai, L.T.; Lan, N.T.; Trinh, D.N.; et al. Preparing three-dimensional graphene aerogels by chemical reducing method: Investigation of synthesis condition and optimization of adsorption capacity of organic dye. *Surfaces and Interfaces* **2021**, *23*, 101023, doi:https://doi.org/10.1016/j.surfin.2021.101023.

7. Wang, Z.; Song, L.; Wang, Y.; Zhang, X.-F.; Yao, J. Construction of a hybrid graphene oxide/nanofibrillated cellulose aerogel used for the efficient removal of methylene blue and tetracycline. *Journal of Physics and Chemistry of Solids* **2021**, *150*, 109839, doi:https://doi.org/10.1016/j.jpcs.2020.109839.
